# Phylogeographic structure of cotton pest *Adelphocoris suturalis* (Hemiptera: Miridae): strong subdivision in China inferred from mtDNA and rDNA ITS markers

**DOI:** 10.1038/srep14009

**Published:** 2015-09-21

**Authors:** Lijuan Zhang, Hu Li, Shujuan Li, Aibing Zhang, Fei Kou, Huaizhu Xun, Pei Wang, Ying Wang, Fan Song, Jianxin Cui, Jinjie Cui, Dawn H. Gouge, Wanzhi Cai

**Affiliations:** 1 Department of Entomology, China Agricultural University, Beijing 100193, China; 2 Maricopa Agricultural Center, University of Arizona, Maricopa, AZ 85138, USA; 3 College of Life Sciences, Capital Normal University, Beijing 100048, China; 4 Key Laboratory of Molluscan Quarantine and Identification of AQSIQ, Fujian Entry-Exit Inspection & Quarantine Bureau, Fuzhou, Fujian 350001, China; 5 Henan Institute of Science and Technology, Xinxiang, Henan 453003, China; 6 Cotton Research Institute, Chinese Academy of Agricultural Sciences, Anyang, Henan 455000, China; 7 Department of Entomology, University of Arizona, Maricopa, AZ 85721, USA

## Abstract

Phylogeographic patterns of some extant plant and vertebrate species have been well studied; however, they are poorly understood in the majority of insects. The study documents analysis of mitochondrial (COI, CYTB and ND5) and nuclear (5.8S rDNA, ITS2 and 28S rDNA) data from 419 individuals of *Adelphocoris suturalis*, which is one of the main cotton pests found in the 31 locations in China and Japan involved in the study. Results show that the species is highly differentiated between populations from central China and peripheral China regions. Analysis of molecular variance showed a high level of geographical differentiation at different hierarchical levels. Isolation-by-distance test showed no significant correlation between genetic distance and geographical distance among *A. suturalis* populations, which suggested gene flow is not restricted by distance. In seven peripheral populations, the high levels of genetic differentiation and the small *N*_*e*_*m* values implied that geographic barriers were more likely restrict gene flow. Neutrality tests and the Bayesian skyline plot suggested population expansion likely happened during the cooling transition between Last Interglacial and Last Glacial Maximum. All lines of evidence suggest that physical barriers, Pleistocene climatic oscillations and geographical heterogeneity have affected the population structure and distribution of this insect in China.

Molecular phylogeographic studies provide valuable insights for exploring specific genetic structure, geographic modes, prevalent time scales, and demographic history^1^. Particularly, a large amount of evolutionary information is stored in cytoplasmic and nuclear genomes, from which population structure and demographic history of a species can be inferred. In addition, fossil and paleogeographic evidence provides important insights to explore the evolutionary information. Genetic variability is considered to be the foundation of evolution, and it can be affected by mutation rates, genetic drift, effective population size and gene flow^2^. The extent of geographic variation may result in local genetic differentiation and genetic homogeneity. Mutation and genetic drift due to finite population size, and natural selection favoring adaptations to local environmental conditions may lead to the genetic differentiation of local populations; and the gene flow may lead to genetic homogeneity among local populations^3^. There are many important factors affecting geographic variation, including local ecological conditions, dispersal ability^4^, climatic change^5^, physical barriers, and complex topography^6^.

The influence of physical barriers on genetic structure has been investigated in seabirds (*Sula leucogaster*, *S. sula*). Morris-Pocock *et al.*^7^ suggested that Isthmus of Panama and the periodic emergence of the Sunda and Sahul Shelves in Southeast Asia served as physical barriers that restricted gene flow among ocean basins. The effects of environmental variables and environmental factors on the population genetic structure have been studied in some species. Huang *et al.*^8^ proposed temperature and rainfall were main factors influencing the genetic structure of the chukar partridge (*Alectoris chukar*). Recent phylogeographic studies indicated that organisms were capable of responding to climate changes and adapting to different climatic conditions^9^. For example, during the Pleistocene, China was a mosaic of mountains with a height lower than 2 kilometers (km) and characterized by a relatively mild Pleistocene climate^10^, potentially hosting microclimatic zones capable of supporting diverse habitats in a relatively stable status. Therefore, multiple glacial refugia may have been available to organisms in China^11^. Hewitt^12^ proposed that rapid expansion from refugia populations would result in lower genetic diversity in the more recently colonized places, whereas the refugia populations would be less affected by climatic changes and would be more genetically diverse. Phylogeographical studies have only recently begun in China. For example, molecular markers have been used to identify independent refugia and track the colonization routes for the black-spotted frog *Pelophylax nigromaculata*^13^, and to illustrate population genetic structure for *Chilo suppressalis*^14^.

The black striped plant bug, *Adelphocoris suturalis* (Jakovlev, 1882) (Hemiptera: Miridae), is a widespread pest in East Asia (e.g. China, Korea, and Japan). It is a major pest on cotton and several other crops in China^15^, while it is considered as one of the most serious alfalfa pests in Japan^16^. As a highly polyphagous insect, *A*. *suturalis* can attack a variety of plants (~115 species), primarily agricultural crops (e.g. cotton, vegetables and fruit crops), pastures and weeds. It causes significant economic losses, and reduces yields and qualities of crops. In addition, this insect may easily reach critically important thresholds, switch host crops, or experience geographic spread because of its great tolerance to climate change^17^, high population growth rate^18^, and strong dispersal capacity^19^. Lu *et al.*^19^ reported that the maximum flight distance reached 82.7 kilometers and sustained flight for 7.6 hours for the bug at the temperatures 20–23 °C and 64–68% relative humidity. Previous studies on this pest were mainly focused on its morphology, ecology and management^16^,^19^,^20^. However, its phylogeography and population structure remains poorly understood.

In this study, the geographical pattern of genetic variation for this species is assessed using three mitochondrial genes (mtDNA) and the nuclear internal transcribed spacer (rDNA ITS) region. The main goals of this study were to (i) analyze the genetic structure and phylogeography of *A. suturalis*; (ii) examine the geographical pattern of haplotypes; (iii) infer the demographic history of *A. suturalis*. The genetic diversity was analyzed along with the levels of genetic variation within and among *A. suturalis* populations. Possible factors that affect the genetic variation were assessed and a reconstruction of the demographic history of *A. suturalis* populations developed.

## Results

### Genetic diversity and structure

A final combined mitochondrial dataset included 2046 bp of protein-coding regions (COI: 603 bp, CYTB: 789 bp and ND5: 654 bp). No insertions or deletions were detected among these fragments. Ninety-five haplotypes (including 70 unique haplotypes) and 107 polymorphic sites (including 58 parsimony sites and 49 singleton sites) were found among all samples (Table S1). Average number of nucleotide differences (K) ranged from 1.086 (GY) to 18.000 (SY) with an average of 6.820 (Table 1). Haplotype diversities (*Hd*) within populations ranged from 0.619 to 1.000 with an average of 0.826 with nucleotide diversities (π) from 0.001 to 0.009.

**Figure 1:**
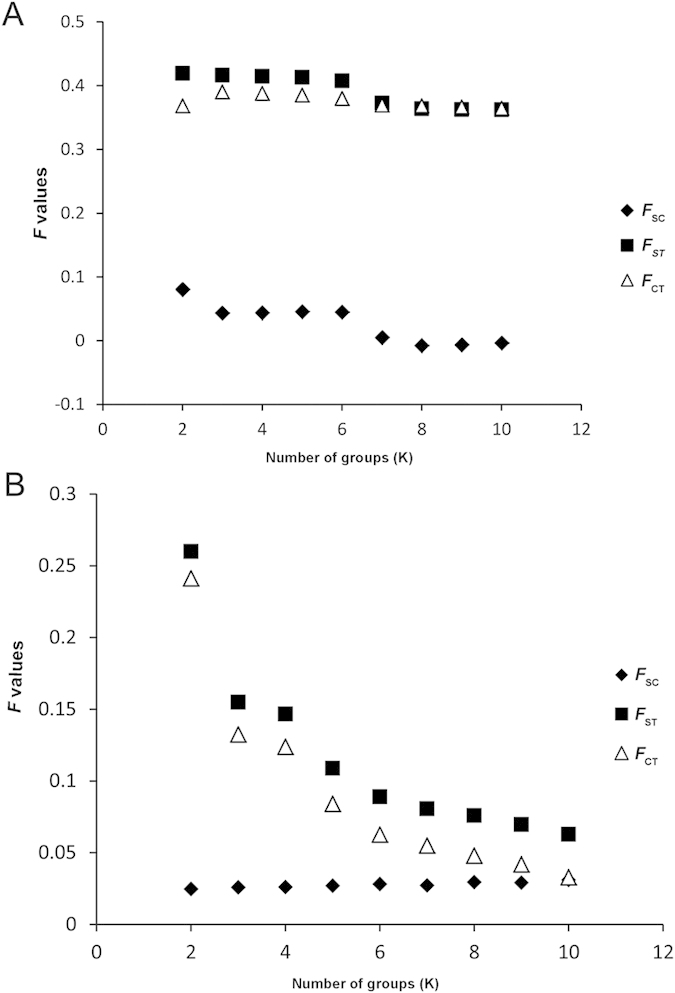
Fixation indices correspond to the number of groups (K) defined by SAMOVA analysis based on mtDNA (A) and ITS (B) data.

SAMOVA analysis showed a distinct increase in *F*_CT_ from *K* = 2 to 3, and a slight decrease of *F*_CT_ values from *K* = 4 to 10. When *K* = 3, *F*_CT_ was significant and up to the largest values (*F*_CT_ = 0.390, P < 0.001); *K* ≥ 4, the population structure disappeared with a single population assigned to a group (Fig. 1A). Therefore, *K* = 3 was used as the population assignment scheme and predicted three groups corresponding well to three geographic regions. The first group (HJ) was the single population from Japan. The second group included 13 populations (ZJ, XC, HS, JJ, YY, XY, NY, SL, PY, CD, QJ, XX, WN) from Central China and North China Plain, one population (WZ) from Northwestern Plateau and one population (SY) from Northeastern China Plain. As most populations of the second group were at the relative central location of *A. suturalis*’s distribution, corresponding to central China geographic regions simultaneously, this group was named the central China group (CC). The third group included the remaining 15 populations, of which 11 populations (ZHZ, LX, CX, BB, JYC, ACZ, XCH, LN, GY, SH, TL, DZ, LF, BJ, TJ) were from east coast, Northwestern China Plateau, Southwestern China Plateau, Northeastern China; four populations from North China Plain (DZ, LF, BJ, TJ). Most populations located in the peripheral provinces of China, which were also at the peripheral location of pest distribution; therefore, this group was defined as the peripheral China group (PC).

Pairwise *F*_ST_ value between PC and CC was *F*_ST_ = 0.380, P < 0.001; between HJ and PC was *F*_ST_ = 0.575, P < 0.001 and between HJ and CC was *F*_ST_ = 0.356, P < 0.001, respectively. These pairwise *F*_ST_ values suggested that the three defined groups were significantly differentiated. Some populations from the peripheral China group showed high *F*_ST_ values (e.g. GY, CX, SH, ZHZ), indicating that a strong natural barrier to gene flow may exist in these populations (Table S2). The three-level AMOVA test also showed significant genetic differentiation between defined groups (39.05%; *F*_CT_ = 0.39, P < 0.001) and the two-level AMOVA analysis revealed the significant differentiation among populations (29.36%, *F*_ST_ = 0.29, P < 0.001) (Table S3). IBD test showed no significant correlation between genetic differentiation (*F*_ST_) and geographical distance (R^2^ = 0.0643, P = 0.99) among populations, and indicated that gene flow was not restricted by distance (Fig. S1).

For ITS region, 406 successful sequences were used in the subsequent alignment analysis. The length of ITS sequence alignment was 1303 bp [including 5.8S (123 bp), ITS2 (1096 bp) and 28S (84 bp)]. 130 haplotypes (including 109 unique haplotypes) and 125 polymorphic sites were found when gaps were treated as fifth state (Table S4). 125 haplotypes and 122 polymorphic sites were found when gaps were treated as missing. High haplotype diversities (*Hd*) were observed in dataset (ranging from 0.5824 to 1.0000 with an average of 0.857) (Table S5).

The SAMOVA analyses revealed a distinct decrease in *F*_CT_ from *K* = 2 to 3, and *F*_CT_ values continue to decrease from *K* = 4 to 10. When *K* > 2, the population structure disappeared with a single population assigned to a group (Fig. 1B). The incongruence of mitochondrial versus nuclear dataset indicated that mtDNA had a relatively high variability and a rapid evolutionary rate compared to the nuclear gene. AMOVA analysis revealed significant genetic differentiation among populations (*F*_ST_ = 0.030, P < 0.001) (Table S3).

### Genetic barriers and gene flow

**Figure 2:**
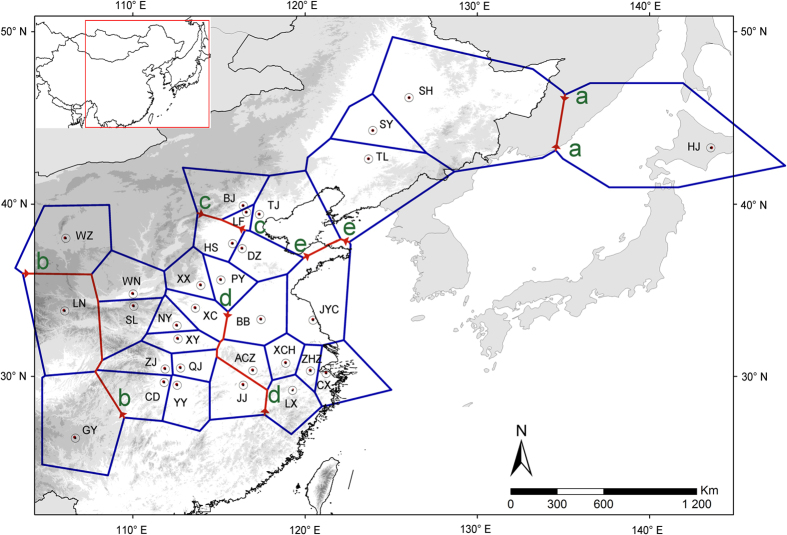
Genetic barriers predicted by BARRER based on mtDNA data. The genetic barriers are shown in red lines with arrows labeled from ‘a’ to ‘e’. Map was generated from http://ngcc.sbsm.gov.cn/ (Data of access: 18/03/2015).

The genetic discontinuity analysis performed by BARRIER on the mtDNA dataset suggested that the hidden genetic barriers were correlated to genetic abruption. The five red lines (shown from ‘a’ to ‘e’ in Fig. 2) separated Japan populations and some peripheral China populations (including HJ, GY, LN, LF, BJ, eastern coastal populations) from central China populations suggested the existence of genetic barriers between central China and peripheral China populations, and between the Japanese populations and Chinese populations. However, northeastern populations were not isolated from central populations, suggesting the existence of certain gene flow between the lineage and central China (Fig. 2).

Similar to the mtDNA results, the analyses of ITS region showed that populations of HJ, GY, LN and BJ were separated from central China populations (shown from ‘a’ to ‘i’ in Fig. S2). However, populations from eastern coastal China were not separated from other central China populations, indicating that there was gene flow among some eastern coastal populations and central populations. The red line ‘g’ separating the CD population from central populations suggested the presence of physical barriers. The red line ‘c’ separating the SH population from northeastern populations implies that different lineages existed in the northeastern region (Fig. S2).

MIGRATE analyses indicated high levels of gene flow among populations, with the average value of 834.9 and the largest value of 1000.0. Asymmetrical gene flow was observed in 44 out of 930 pairwise comparisons. This was supported by non-overlapping 95% confidence intervals for the estimate of migration parameter (*M*) for each population (denoted in bold in Table S6). For example, *M* is estimated to be 1000.0 from populations BB to XX, but only 12.0 from XX to BB. Asymmetrical gene flow happened from southeastern China (e.g. BB, LX and ACZ) to adjacent and northern China regions (e.g. XX, XY, CX), from northern China (e.g. HS, TJ) to northeastern China regions (e.g. SH, TL), and from Mt. Qinling regions (e.g. WN, PY, XY) to other regions (e.g. WZ, QJ, HS).

The seven populations (ZHZ, CX, GY, LN, SH, TL, BJ) showed much higher levels of gene flow than the rest of the studied populations. Interestingly, when translated *M* values into effective migrants per generation (*N*_*e*_*m*), the *N*_*e*_*m* values were much smaller (<2 migrants per generation) in the above seven populations compared to other populations (Table S7). The results suggested that inbreeding within a population was more frequent and severe among these populations. In addition, the low effective population size (*θ*) observed in these seven populations implied the potential effect of a small size founder population (Table S6).

### Phylogenetic analysis and network construction

Based on mtDNA data, the BI tree of haplotypes formed two clades supported by 100% and 67%, respectively (Fig. S3). Clade A comprised populations from central China and Japan, most Japanese haplotypes were grouped together and separated from central China with a support value of 100%; Clade B comprised of populations from peripheral China. ML tree of haplotypes produced two clades, which were concordant with the topology of the BI tree, with lower bootstrap values. For the ITS region, the BI tree and ML phylogenetic tree of haplotypes (gaps as “5th” state) covered two clades with relatively lower support values (Fig. S4). Clade A included all populations from central China, and clade B included all populations from peripheral China. Japanese haplotypes were assigned into the two Chinese clades.

**Figure 3:**
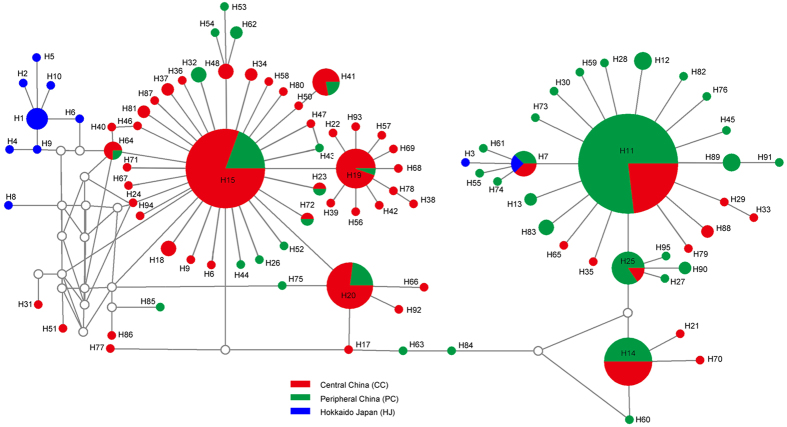
Haplotype network of *Adelphocoris suturalis* based on mtDNA data. The circle size of haplotype denotes the number of observed individuals. Colors correspond to different regions. White circles represent intermediate haplotypes not observed.

In the haplotype network, the ancient haplotypes generally should be located at the center of the network and have more widespread distributions, while the recent haplotypes should be at the tips of the network and be localized geographically^21^. The two most frequent mtDNA haplotypes in the study were: H11, which was the major haplotype of peripheral China, which comprise of 121 individuals from 27 populations; H15 was the major haplotype of central China, which included 77 individuals from 24 populations (Fig. 3). The other haplotypes: H14, H19 H20 and H25, comprised of 28, 19, 26 and 13 individuals from 15, 10, 14 and 7 populations, respectively. The star-shaped network suggested that most haplotypes differed from H11 and H15 by only a few mutations, respectively. The network suggested that *A. suturalis* experienced at least two population expansion events. The central China populations from Mt. Qinling (WN, SL, XC, XX, PY and HS) had a relatively high proportion (71.76%) of shared haplotypes (H11, H15, H14, H19, H20 and H25), whereas other populations (NY, XY, ZJ, QJ, CD, YY and JJ) had 60% shared haplotypes. This would suggest that populations from Mt. Qinling region might have established relatively early in these areas. Most Japanese haplotypes have the long-branch length in network suggesting that Japanese populations may have remained stable after colonization. That more haplotypes were found in central China than peripheral China suggested the existence of more relatively suitable habitats in central China.

**Figure 4:**
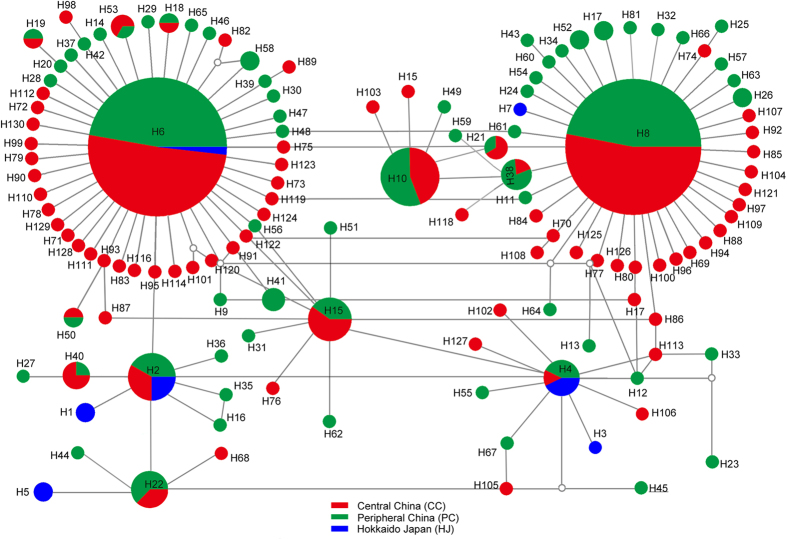
Haplotype network of *Adelphocoris suturalis* based on ITS data. The circle size of haplotype denotes the number of observed individuals. Colors correspond to different regions. White circles represent intermediate haplotypes not observed.

Similar to the results of mtDNA, the ITS network (gaps as “5th” state) presented two of the most frequent haplotypes H5 and H8 (Fig. 4). H5 was the most frequent haplotype in central China region, which was common to 110 individuals from all study populations (i.e. 31 geographic populations); the other primary haplotype H8, common to 98 individuals from 27 populations. The shared haplotype H10 include 18 individuals from 10 populations. The star-like network observed in Fig. 4 also would support the theory that *A. suturalis* experienced population expansion events.

### History demography

Demographic changes of *A. suturalis* populations were inferred using neutrality tests. When all samples were pooled as one group or defined into three respective groups (PC, CC, HJ), Fu’s Fs, Fu and Li’s D^*^ and Fu and Li’s F^*^ were all significantly negative. However, the significantly negative values were not observed in the Japanese population (Table S8). This implied that the populations from Chinese regions experienced population expansion events and the Japanese population is a constant population that did not experience population expansion. In the peripheral China group, population expansion time (τ) was as follows: τ = 187.527 for the Southeastern China subregion (comprised of BB, JYC, ACZ, XCH, ZHZ, LX and CX), τ = 21.242 for the Northern China subregion (DZ, LF, BJ and TJ), τ = 2.270 for the Northeastern China subregion (SH, TL) and τ = 2.297 for the Southwestern China subregion (GY, LN). Compared to the Northern China and Northeastern China subregion, populations from the Southeastern China subregion began to expand much earlier.

**Figure 5:**
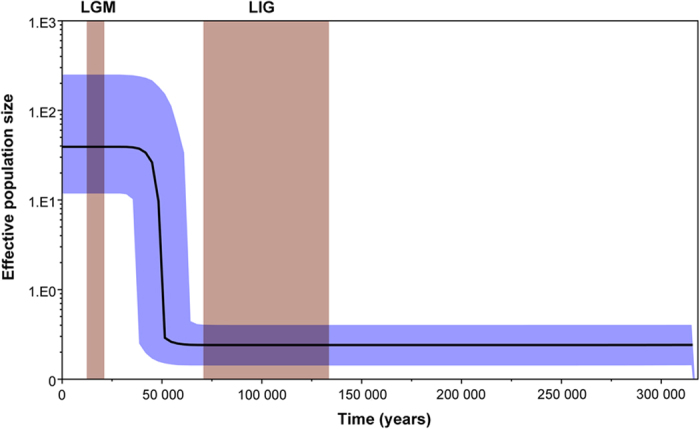
Demographic history of *Adelphocoris suturalis* reconstructed using Bayesian skyline plot based on mtDNA data. X-axis is the timescale before present, and Y-axis is the estimated effective population size. Solid curves indicate median effective population size; the shaded range indicates 95% highest posterior density intervals. LGM represents Last Glacial Maximum, and LIG represents the Last Interglacial.

*A. suturalis* may experience a rapid population growth based on the demographic history reconstruction using Bayesian skyline plot. The most recent common ancestor (TMRCA) of the mitochondrial clades ranged from 67700 to 359200 years before present. The beginning of rapid population growth of *A. suturalis* was around 50000 years ago, and then the demographic trend plateaued about 25000 years ago [i.e. population expansion occurred during the transition from Last Interglacial (LIG) to Last Glacial Maximum (LGM)] (Fig. 5).

## Discussion

Pairwise *F*_ST_ values and AMOVA analysis showed significant genetic differentiation (*F*_ST_) among three defined groups based on the analyses of mtDNA. However, the SAMOVA analyses showed a lack of genetic structure among *A. suturalis* populations using the ITS dataset. It is suggested that the cause of homogeneity in *A. suturalis* is the high level of gene flow. Compared to protein-coding mtDNA markers, non-coding ITS markers are expected to be more sensitive at detecting gene flow among interbreeding populations^22^.

The large number of significant pairwise *F*_ST_ values among groups support population differentiation (Table S2). Physical barriers and ecological climate factors may play an important role to prevent the gene flow among groups. The Mt. Hengduan, Mt. Huang, Mt. Tianmu, Mt. Changbai, Three Gorges mountain region and Yungui Plateau might have acted as substantial geographical barriers, similar physical separations have caused similar results found in populations of the semi-aquatic bug *Microvelia douglasi*^23^ and Chinese Hwamei *Leucodioptron canorum*^24^. For the high *F*_ST_ values between Japanese and Chinese populations, the Bohai Sea, Yellow Sea, East China Sea and Sea of Japan served as geographic barriers and likely playing an important role in the current genetic structure. These seas have also been found to act as geographic barriers reinforcing population structure in terrestrial species, such as *Reticulitermes speratus*^25^ and *Quercus mongolica* var*. crispula*^26^.

The low genetic differentiation within groups implied that population expansion and relatively active dispersal capacity might result in increased gene flow within the group. Sampling locations throughout Chinese temperate and subtropical regions were based on the climate division in China^27^. Ting^18^ reported on the important effects of temperature and humidity on the geographic distribution and development of *A. suturalis*. Therefore, distinct ecological climates in different regions might also contribute to the limitation of gene flow among regions. Compared to central China regions, the peripheral China regions had predominant small-scale host plant plantations, diverse climates and complicated topography that could result in relatively high genetic differentiation between populations.

GY and LN located in southwestern China are separated from other populations due to specific topography. High haplotype diversity was found in these two populations, which is consistent with the statement of Petit *et al.*^28^ that a species usually bear high genetic diversity in suitable habitats. *A. suturalis* samples were collected in Guiyang, an important part of Yungui Plateau, and Longnan, the only area belonging to Yangtze River valley in Gansu Province. The region is located in the ‘intermediate belt’ of southwestern China^29^ with particularly diverse topography and climate, which has become a biodiversity hot spot^30^. The Yungui Plateau may act as a physical barrier to restrict gene flow, which has recently been reported to affect the genetic structure of plant species such as *Cephalotaxue oliveri*^31^.

Eastern China and adjacent regions have experienced dramatic changes in palaeo-landscape structure due to sea-level fluctuations during the Quaternary Period^32^. Such changes in sea level alternately separated and joined eastern China, southern Japan, and the Korean Peninsula, and provided abundant opportunities for population fragmentation and allopatric speciation^11^. High genetic differentiation between eastern China populations and central China populations was observed in our data. Indeed, the black spotted frog *Pelophylax nigromaculata* has also shown notable intraspecific phylogeographic structure across eastern China^13^. In this region, three populations CX, ZHZ and LX showed significantly lower haplotype diversity (P < 0.001) than other populations, suggesting that the recent colonization, local suboptimal ecological conditions and physical barriers (e.g. Mt. Huangshan and Mt. Tianmu) might result in a small size of founder population. The genetic data showed that low effective population size (e.g. CX, *θ* = 0.0015; ZHZ, *θ* = 0.0015) in these populations (Table S6). Markert *et al.*^33^ also proposed that populations with the reduced fitness under permissive conditions could result in low genetic diversity.

High genetic differentiation was observed between Beijing (BJ) and other populations. ITS and mtDNA data presented a geographic barrier in this area (Fig. 3 and S2, respectively). The small scale of host plants and lesser occurrence of natural habitat for primary wild host plants of *A. suturalis* may result in a small population size. The genetic data recorded in this study showed low effective population size (*θ* = 0.0014) in Beijing. A genotype differentiation study of *Helicoverpa armigera*, once a key cotton pest, indicated that existence of a very small number of native individuals in Beijing area^34^.

High genetic differentiation between Changde (CD) and other populations was observed. Nuclear data show a barrier separating this population from central China populations (Fig. S2). The interpretation is that the Three Gorges Mountain Region has acted as a physical barrier and may play a role in hindering gene flow between the Changde population and central China populations. The Three Gorges mountain region ranges from 1 km to 1.5 km above sea level. Phylogeographic studies of the *Myricaria* spp. (Tamaricaceae) showed that the region worked as a natural dispersal barrier, which promoted strong population structure^35^.

Tieling (TL), Suihua (SH) and Songyuan (SY) are located in northeastern China which is a mega-diversity region, with complex topography in the latitude of 40-50°N, which includes the Northeast Plain and major mountain ranges, e.g. Mt. Changbai. High genetic differentiation was shown among Tieling, Suihua and other populations, which might be explained by local suboptimal ecological conditions for *A. suturalis*. Suihua is located in the Sanjiang Plain, the largest marshland area of China, and Tieling is located in the Liaohe River Plain in which flooding normally occurs that could influence or delay the development of *A. suturalis*. Haplotype diversity of TL and SH populations are significantly lower than other populations (P < 0.05), except for three eastern China populations (ZHZ, CX, LX). The ITS data showed a geographic barrier separating Suihua from northeastern China populations. The Songhua River might act as a physical barrier separating Suihua from northeastern populations. Songyuan located in Songnen Plain with superior resources is a suitable eco-geographic condition may allow *A. suturalis* to adapt to new habitats quickly under natural selection. Indeed, high genetic diversity further supports this hypothesis.

Selective neutrality, subdivision or expansion can be distinguished by statistical tests of neutrality of mutations. The results of neutrality tests indicated *A. suturalis* populations experienced population expansion, although a mutation rate of 0.0115 in recently diverged haplotypes at the population level is higher than the phylogenetic rates for some species^36^. For *A. suturalis*, the historical population trend inferred by the Bayesian skyline plot seems to fit the climate trend relatively well during the Pleistocene periods. *A. suturalis* started expanding about 50000 years ago, which appeared during the Marine Isotope Stage 3 (MIS3, 27000–60000 years before present). In East Asian, this stage is a warm and humid period with temperatures about 2–5 °C warmer than present^37^ and an enlarged area of increased precipitation in inland China. Based on pollen records, one study suggests the paleoclimate during the last glacial period was the warmest and wettest during the early MIS3^38^. The warmer temperatures and increased precipitation not only contribute to *A. suturalis*’s physiological activity, but also provide suitable conditions for *A. suturalis* in relatively arid regions of Northwest China, increasing the number of habitats in Mainland China. In addition, the study of Jiang *et al.*^38^ indicated that pollen zone B (46000–60600 years before present, correlative to the early MIS3) had the greatest abundances of Cupressaceae, *Tsuga*, Gramineae, Chenopodiaceae and Cyperaceae of the entire section. As Gramineae and Chenopodiaceae are the primary wild hosts of *A. suturalis*, the increase in population size of these species would distinctly improve the fecundity and survival capacity of the insect.

With regard to climate cooling during the LGM period, we expected that profound ecological upheavals would reduce the population size of *A. suturalis*, however, genetic analyses showed that the species’ *N*_*e*_ was not reduced. A similar situation has been found in a rodent *Microtus montanus,* the population size of which decreased at times of climatic oscillations, whereas *N*_*e*_ remained stable throughout the past 2500 years^39^. In theory, the population size could significantly be reduced due to habitat loss and fragmentation, and its *N*_*e*_ can be highly inflated due to other factors that hinder species’ dispersal among subpopulations^40^. Our study provides no evidence for the hypothesis of Li *et al.*^24^ that intermittent gene flow between refugia during warmer interstadials might have resulted in a large effective population of Chinese Hwamei through the LGM period. Here, we suggested that geographic barriers might have hindered dispersal of *A. suturalis* among expanded populations during the warmer interglacial period. However, the biological characteristics of the species, for example, high population growth rate, switching host crops, great tolerance to climate change, and strong dispersal capacity, might have resulted in a large effective population of this species through the LGM period.

The above analyses show that demographic expansion of the bug is concordant with the records of palaeogeography, palaeovegetation and palaeoclimate in the area. However, the geographic origin of *A. suturalis* is not known, but based on the genetic data presented here, it is possible to speculate the region of origin. Genetic data show that the coastline area facing the Yellow Sea, where the bug has been likely established for a longer period of time, to a very large mainland region that has been colonized in the recent years. Population expansion time (mtDNA: Yancheng, JYC, τ = 17.71, Bengbu, BB, τ = 17.11; ITS: Yancheng, JYC, τ = 2.56) and haplotype data (mtDNA: eight haplotypes from JYC, of which four were shared by 31 populations that covering the Chinese and Japanese regions, nine haplotypes of BB of which six were shared by 31 sampling populations; ITS: BB and JYC had a largest number of individuals belong to main haplotype H5 and H8) support the idea that the insect is most likely originated in China’s eastern coast region.

Regarding the recent population expansion into the present range, we suggest that the more recent expansion of primary hosts have provided a new opportunity for *A. suturalis*. Beck & Reese^41^ proposed that insect survivorship, fecundity, growth rate and activity can be affected by the size and quality of hosts. The abundance of hosts in the habitat will increase the survival and fecundity and reduce the mortality. Our sampling efforts and communications with collaborators in northeastern China and northwestern China, with regard to the development of animal husbandry, the main forage used included primary crop hosts of *A. suturalis*, such as *Medicago sativa*, and *Melilotus albus*. Furthermore, the planting area of the most important crop host of *A. suturalis*, the transgenic Bt (*Bacillus thuringiensis*) cotton, has obviously expanded from approximately 10000 ha to more than 3.7 million ha since 1997^42^. Additionally, the Bt cotton can distinctly reduce the need for insecticide sprays, and this low level of selective pressure of pesticide on the insect might be the other reason leading to the sudden demographic expansion. The tree hosts of the insect include *Malus* spp. (*Malus domestica*, *Malus prunifolia*), *Prunus* spp. (*Prunnus armeniaca*, *Prunnus cerasifera*), *Vitis vinifera*, and *Ziziphus jujube*, and the extensive commercial cultivation of these trees in recent years may also contribute to the expansion of the insect territory.

Based on the palaeoclimatic and palaeovegetational evidence as well as genetic data presented, we suggest that two independent refugia might have existed during the Pleistocene for *A. suturalis*. One refugium was the eastern coast of China. Reconstructed LGM vegetation shows that the cool-temperate deciduous forests of today were the major vegetation type within the range of extant *A. suturalis*^43^. The temperate deciduous forests in northern China retain genetic signals of postglacial northward range expansion^44^, for example, a substantial impoverishment of (intra-population) genetic diversity in the direction of a wave-like spread^45^. The data support that the distribution pattern of *A. suturalis* is the result of range expansion from the Southeastern China subregion during the last interglacial period. The fact that populations from Southeastern China subregion began to expand much earlier than other regions, along with the asymmetric gene flow from Southeastern China to other regions, and the relatively high haplotype diversity (e.g. BB, JYC, XCH) all imply that this region might have acted as the ice-age refugium. From this refugium, north- and northeastward expansion of *A. suturalis* along with the expansion of the deciduous forests might have occurred in these regions, which form the current distribution pattern. This distribution pattern of *A. suturalis* was consistent with the phylogeographical pattern observed in deciduous forests within these regions. For example, in the direction of north- and northeastward range expansion, our genetic data showed a substantial impoverishment of genetic diversity in some populations (three southeastern China populations (ZHZ, CX, LX); two northern China populations (BJ, LF); two populations from northeastern China (TL and SH).

We suggest that another refugium might be located at Mt. Qinling (at c. 34°N), in the central distribution region of *A. suturalis* in China. The Mt. Qinling region underwent complex changes in climate and vegetation distributions throughout the last ice age cycles^46^. This region is a mosaic of mountains with a relatively mild Pleistocene climate^10^. The complex landforms and the influences of geological events on this biota are likely to have retained genetic signals of both glacial and inter-/postglacial fragmentation, for example, high genetic diversity among populations. Indeed, Mt. Qinling and adjacent regions acting as refugia has been proven for other species^24^. Our genetic data showed that relatively high proportions of shared haplotypes were observed in populations from the Mt. Qinling region. In addition, asymmetric gene flow from the Mt. Qinling region to other regions further suggest that the Mt. Qinling region might have acted as a refugium for the species during the Pleistocene.

Regarding the phylogeography of *A. suturalis* between Japanese and Chinese fauna realms, we did not draw substantial conclusions from our statistical data, due to sampling only the population from Japan, and a lack of sampling populations and sufficiently available sequences from adjacent countries (e.g. Korea, Russia). More samples from adjacent countries need to be collected to study the phylogeography of the species in these countries.

## Methods

### Sample collection

**Figure 6:**
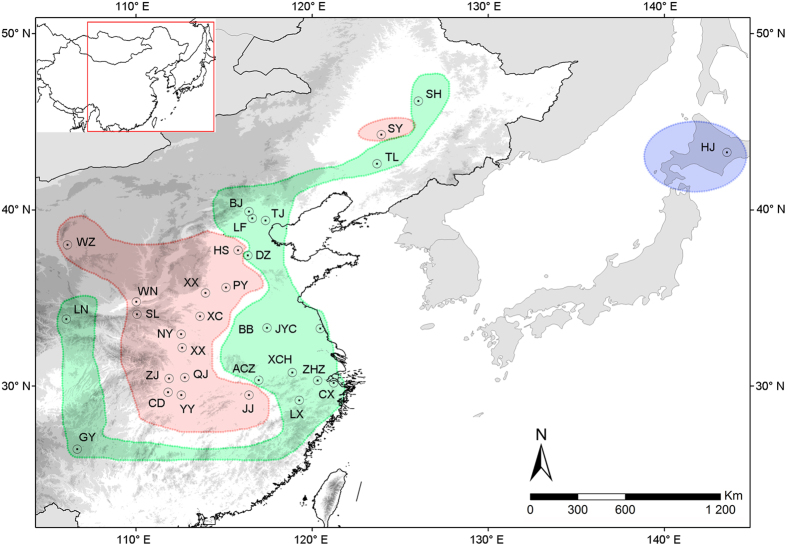
Map of sampling localities of the *Adelphocoris suturalis*. Different colors showed three groups defined by SAMOVA based on mitochondrial data (red color represents central group; green color represents peripheral group; blue color represents Hokkaido Japan group). Map was generated from http://ngcc.sbsm.gov.cn/ (Data of access: 18/03/2015).

A total of 419 individuals were collected from 30 locations in China and one location in northern Japan between 2011 and 2013 (Table S9 and Fig. 6). The linear geographical distance among the sampling locations ranges from 45.79 to 3813.15 km with an average of 978.14 km. Voucher specimens are deposited at the China Agricultural University, Beijing, China.

### DNA extraction, amplification and sequencing

Total genomic DNA was extracted from single adult insect using the DNeasy Blood and Tissue Kit (Qiagen, Hilden, Germany). The abdomen of samples was removed prior to DNA extraction. Fragments of three mtDNA genes (COI, CYTB and ND5) and nuclear ITS region (including the complete ITS2 with partial 5.8S and 28S rDNA regions) were used as molecular markers for this study.

The PCR amplifications of mitochondrial genes were performed using specific designed primers, while the ITS region was amplified using primer pairs P1^47^ and 28Z^48^ (Table S10). Amplifications were conducted in 25 μL volumes containing 5 μL of 10 × PCR buffer, 0.2 mM each dNTP, 0.25 μM each of primer, 0.25 unit Taq DNA polymerase (Takara Biotechnology, Dalian, China) and 2 μL of template DNA under the following conditions: initial denaturation of 3 min at 94 °C, followed by 35 cycles with 30 s at 94 °C, 30 s at 54–58 °C and 1 min at 72 °C, and a final extension step at 72 °C for 5 min.

Purified PCR products were sequenced in both directions with the ABI 3730xl DNA Analyzer at Ruibo Biotechnology Co., Ltd (Beijing, China). All sequences have been deposited in GenBank under accession numbers KM981463-KM981497 for COI, KM981498-KM981628 for ITS2 plus 28S and 5.8S, KM981629-KM981667 for ND5, and KM981668-KM981704 for CYTB.

### Genetic diversity and structure

Sequences of ITS and mtDNA markers were aligned independently using ClustalW implemented in Mega 6.0^49^ with default parameters. Alignment of nucleotide sequences of the mitochondrial protein-coding genes (COI, CYTB and ND5) was inferred from the amino acid alignment and examined for the presence of stop codons and other indicators that they were nuclear copies. ITS2 sequences were delimitated and identified based on hidden Markov models (HMMs)^50^ on http://its2.bioapps.biozentrum.uni-wuerzburg.de/. The number of polymorphic sites (*S*), haplotype diversity (*Hd*), nucleotide diversity (π), average number of nucleotide differences (K) and the number of haplotypes (Ht) were calculated using DnaSP 5.0^51^ or Arlequin 3.5^52^.

The SAMOVA 1.0 program^53^ was used to define the genetic structure of populations with *K* values ranging from 2 to 10 and the 100 independent simulated annealing processes. This method is based on a simulated annealing process that maximizes the proportion of total genetic variance due to differences between groups, and leads to the identification of genetic barriers between the groups (the values of fixation indices of genetic differentiation: among populations within groups, *F*_SC_; among groups, *F*_CT_; between populations among groups, *F*_ST_). One study reported on the configurations *K* with one or more single population groups fails to produce the group structure^54^. Therefore, the highest *F*_CT_ that did not contain any single population group could be used to identify the groups of populations.

The genetic differentiation among populations and defined groups were further estimated based on pairwise *F*_ST_ values, and population genetic structure was analyzed based on the analysis of molecular variance (AMOVA) in Arlequin. Arlequin was also used to calculate the genetic distance (*F*_ST_) matrix to test for the presence of Isolation-by-distance (IBD) in the dataset. Google Earth (http://earth.google.com) was used to estimate the linear geographic distance (km) between the sampling locations. The significance was tested with the Mantel test employing 1000 randomizations in IBDWS 3.23^55^.

### Genetic barriers and gene flow

In order to identify the hidden genetic barriers resulting from microevolutionary processes (such as genetic drift, gene flow and natural selection), the program BARRIER 2.2^56^ with Monmonier’s algorithm was used. Pairwise migration rate between populations was estimated by the MIGRATE 3.6^57^. A Bayesian search strategy was used to calculate mutation-scaled population size (*θ* = *xN*_*e*_*μ*, where *x* is the inheritance parameter; *N*_*e*_ is the effective population size; *μ* is the mutation rate per site per generation) and mutation-scaled migration rate (*M* = *m*/*μ*, where *m* is the immigration rate). The effective number of migrants of each population per generation (*N*_*e*_*m*) is *θM*. Heating chain was set: 1.0, 1.5, 3.0, and 6.0. Four independent runs of 20000000 generations were conducted to examine the consistency of output results with the first 10000 generations discarded.

### Phylogeographic analysis and network construction

Two datasets of mitochondrial and nuclear haplotypes were analyzed under both Bayesian inference (BI) and maximum likelihood (ML) analysis using MrBayes 3.2^58^ and PhyML 3.0^59^, respectively. *A. lineolatus* was used as an outgroup due to its close relationship with *A. suturalis*. ML analyses with 1000 bootstrap replicates were performed in PhyML. For MrBayes analyses, separate partitions were created for each gene in the mitochondrial dataset with the best-fit model, which was determined using jModelTest 2.1^60^ for each gene. The best-fit model was HKY+I for COI, CYTB and ND5. The GTR+G model was utilized for ITS haplotypes data set. Two simultaneous runs of 10 million generations were performed for the dataset and trees were sampled every 1000 generations, with the first 25% discarded as burn-in. Markov chain stationary was considered to be reached when the average standard deviation of split frequencies was below 0.01^61^. The haplotype networks of ITS and mtDNA data were constructed in Network 4.6^62^ with the median-joining algorithm.

### Historical demography

To investigate demographic history of *A. suturalis*, population expansion time (τ) and neutrality analysis of Fu’s Fs^63^ and P values were estimated using the software Arlequin. Neutrality analyses of Fu and Li’s F^*^, Fu and Li’s D^*^^64^ and P values were calculated using DnaSP 5.0. Fs seems to be a promising test for detecting population growth and genetic hitchhiking^63^. Under the assumption of neutrality, the population expansion produces a significantly negative Fs value^63^. When a population size is constant, Fu’s Fs is considered to be nearly zero, whereas significantly positive values indicate processes such as population subdivision or recent population bottleneck. Fu and Li’s tests appear to be the best for detecting background selection^63^.

The Bayesian skyline plot, a method for estimating past population dynamics from molecular sequences, implemented in BEAST 1.6^65^, was performed to estimate divergence time using a Yule process model. The relaxed uncorrelated lognormal molecular clock was applied with a mutation rate of 0.0115/Ma based on appropriately 2.3% sequences divergence per million years for COI^66^, which has been widely applied to estimate divergence times for insect mitochondrial DNA. HKY+I model was employed for COI, CYTB and ND5 genes. The MCMC chain length was set for 100 × 10^6^ generations with sampling every 10000 generations. The results of the calculation were checked in Tracer 1.4^65^. TreeAnnotator 1.6 (http://beast.bio.ed.ac.uk/TreeAnnotator/) was used to summarize sample information after discarding the first 10% of the trees.

## Additional Information

**How to cite this article**: Zhang, L. *et al.* Phylogeographic structure of cotton pest *Adelphocoris suturalis* (Hemiptera: Miridae): strong subdivision in China inferred from mtDNA and rDNA ITS markers. *Sci. Rep.* **5**, 14009; doi: 10.1038/srep14009 (2015).

## Supplementary Material

Supplementary Information

## Figures and Tables

**Table 1:** Genetic diversity statistics for mtDNA data in *Adelphocoris suturalis* sampling populations.

Population	Locality	*S*	Ht	*Hd*	π	K
HJ region						
HJ	Hokkaido, Japan	39	10	0.8571	0.0043	8.8761
PC region						
ZHZ	Hangzhou, Zhejiang, China	18	5	0.5619	0.0014	2.8571
LX	Lanxi, Zhejiang, China	20	6	0.7143	0.0035	7.0667
GY	Guiyang, Guizhou, China	5	6	0.8000	0.0005	1.0857
SH	Suihua, Heilongjiang, China	17	5	0.7429	0.0014	2.8191
BB	Bengbu, Anhui, China	21	9	0.8476	0.0040	8.2667
JYC	Yancheng, Jiangsu, China	24	8	0.8667	0.0044	8.8952
ACZ	Chizhou, Anhui, China	21	7	0.8191	0.0042	8.5143
DZ	Dezhou, Shandong, China	21	8	0.8762	0.0045	9.2762
TL	Tieling, Liaoning, China	16	4	0.6191	0.0027	5.6000
LF	Langfang, Hebei, China	20	7	0.7714	0.0039	8.0191
CX	Cixi, Zhejiang, China	17	3	0.2571	0.0011	2.2667
BJ	Haidian, Beijing, China	17	4	0.6381	0.0018	3.7524
LN	Longnan, Gansu, China	18	5	0.8222	0.0033	6.6444
XCH	Xuancheng, Anhui, China	18	5	0.9333	0.0048	9.8000
TJ	Baodier, Tianjin, China	—	1	—	—	—
CC region						
ZJ	Zhijiang, Hubei, China	19	8	0.8762	0.0016	3.1810
XC	Xuchang, Henan, China	19	6	0.8381	0.0029	6.0000
HS	Hengshui, Hebei, China	22	9	0.8857	0.0031	6.3048
JJ	Jiujiang, Jiangxi, China	25	8	0.8667	0.0038	7.7333
YY	Yueyang, Hunan, China	23	8	0.8952	0.0040	8.1905
XY	Xiangyang, Hubei, China	23	10	0.9333	0.0036	7.2571
NY	Nanyang, Henan, China	19	7	0.8762	0.0025	5.1429
WZ	Wuzhong, Ningxia, China	19	6	0.6476	0.0031	6.3048
SL	Shangluo, Shanxi, China	8	7	0.8000	0.0008	1.5619
PY	Puyang, Henan, China	23	9	0.8857	0.0045	9.1048
CD	Changde, Hunan, China	23	10	0.9238	0.0035	7.2000
QJ	Qianjiang, Hubei, China	21	8	0.8667	0.0041	8.4381
XX	Xinxiang, Henan, China	19	8	0.8952	0.0041	8.3429
WN	Weinan, Shanxi, China	19	6	0.8889	0.0040	8.1111
SY	Songyuan, Jilin, China	18	2	1.0000	0.0088	18.0000
Total		107	95	0.8722	0.0044	8.9093

*S*, number of segregating sites; Ht, the number of haplotypes; *Hd*, haplotype diversity; π, nucleotide diversity; K, average number of nucleotide difference.
