# Supplementary material for: Phylogeographic structure of cotton pest Adelphocoris suturalis (Hemiptera: Miridae): strong subdivision in China inferred from mtDNA and rDNA ITS markers
Source: Sci Rep. 2015 Sep 21;5:14009. doi: 10.1038/srep14009 (PMC4585665; doi:10.1038/srep14009)

Supplementary Information

Phylogeographic structure of cotton pest *Adelphocoris suturalis* (Hemiptera: Miridae): strong subdivision in China inferred from mtDNA and rDNA ITS markers

Lijuan Zhang[1,6]*, Hu Li[1]*, Shujuan Li[2], Aibing Zhang[3], Fei Kou[1], Huaizhu Xun[1], Pei Wang[4], Ying Wang[5], Fan Song[1], Jianxin Cui[5], Jinjie Cui[6], Dawn H. Gouge[7] and Wanzhi Cai[1§]

[1]Department of Entomology, China Agricultural University, Beijing 100193, China

[2]Maricopa Agricultural Center, University of Arizona, Maricopa, AZ 85138, USA

[3]College of Life Sciences, Capital Normal University, Beijing 100048, China

[4]Key Laboratory of Molluscan Quarantine and Identification of AQSIQ, Fujian Entry-Exit Inspection & Quarantine Bureau, Fuzhou, Fujian 350001, China

[5]Henan Institute of Science and Technology, Xinxiang, Henan 453003, China

[6] Cotton Research Institute, Chinese Academy of Agricultural Sciences, Anyang, Henan 455000, China

[7]Department of Entomology, University of Arizona, Maricopa, AZ 85721, USA

* These authors contributed equally to this work.

§Correspondence and requests for materials should be addressed to W. C. (caiwz@cau.edu.cn).

Supplementary Table S1. Haplotype frequency by population based on combined mitochondrial DNA sequences of *Adelphocoris suturalis*.

Haplotype	Haplotype frequency by population																														
frequency	ZHZ	ZJ	XC	LX	GY	HS	SH	JJ	YY	BB	XY	NY	WZ	JYC	SL	ACZ	DZ	PY	TL	CD	LF	CX	QJ	XX	BJ	WN	LN	XCH	HJ	SY	TJ
Hap_1																													6		
Hap_2																													1		
Hap_3																													1		
Hap_4																													1		
Hap_5																													1		
Hap_6																													1		
Hap_7										1				1			2							2					1		
Hap_8											1																		1		
Hap_9																													1		
Hap_10																													1		
Hap_11	10		3	8	6	2	7	3	3	6	2	2	1	5		6	5	5	9	1	7	13	3	1	8	1		2		1	1
Hap_12	1				3																										
Hap_13	2																														
Hap_14	1								2	2	1		2	3		1	1	1	1	2	1		1	4	5						
Hap_15	1	5	5	2		5	1	5	4	1	3	4	9	2	6	2		1	3	1	3		5	3	1	3	2				
Hap_16		1																													
Hap_17		1																													
Hap_18		2													1																
Hap_19		2	3			1				1	1	3			1			1		4						2					
Hap_20		2	2			1		1	2	1	3				4	3	1			2		1	2			1					
Hap_21		1																													
Hap_22		1																													
Hap_23			1	1																											
Hap_24			1																												

Hap_25	2	3		3						1				2	1	1	
Hap_26	1																
Hap_27	1																
Hap_28		1															
Hap_29		1															
Hap_30		1															
Hap_31			1														
Hap_32			2														1
Hap_33			1														
Hap_34					2												
Hap_35					1												
Hap_36					1												
Hap_37					1					1							
Hap_38					1												
Hap_39						1											
Hap_40						1											
Hap_41						1		1	3	1		2	1				
Hap_42						1											
Hap_43							1										
Hap_44							1										
Hap_45							1										
Hap_46								1									
Hap_47								1									
Hap_48								1					2				
Hap_49									1								
Hap_50									1								
Hap_51									1								
Hap_52											1						

Hap_53	1									
Hap_54	1									
Hap_55	1									
Hap_56		1								
Hap_57		1								
Hap_58		1								
Hap_59			1							
Hap_60			1							
Hap_61			1							
Hap_62				2						
Hap_63				1						
Hap_64				1	2					1
Hap_65					1					
Hap_66					1					
Hap_67						1				
Hap_68						1				
Hap_69						1				
Hap_70						1				
Hap_71						1				
Hap_72							1			1
Hap_73							1			
Hap_74							1			
Hap_75							1			
Hap_76								1		
Hap_77									1	
Hap_78									1	
Hap_79									1	
Hap_80										1

Hap_81																			2						
Hap_82																				1					
Hap_83		3																							
Hap_84																							1		
Hap_85																							1		
Hap_86																							1		
Hap_87																					1				
Hap_88																					2				
Hap_89																						4			
Hap_90																						2			
Hap_91																						1			
Hap_92																									1
Hap_93	1																								
Hap_94	1																								
Hap_95		1																							

Supplementary Table S2. Pairwise *FST* values for 31 populations of *Adelphocoris suturalis* based on mtDNA data.

Population	HJ	ZHZ	LX	GY	BB	JYC	ACZ	DZ	TL	LF	CX	BJ	SH	XCH	LN	TJ	ZJ	XC	HS	JJ	YY	XY	NY	WZ	SL	PY	CD	QJ	XX	WN	ST
HJ	0.000																														
ZHZ	**0.663*****	0.000																													
LX	**0.487*****	0.065	0.000																												
GY	**0.724*****	0.003	**0.173***	0.000																											
BB	**0.432*****	**0.140***	-0.041	**0.260*****	0.000																										
JYC	**0.420*****	**0.160****	-0.024	**0.276*****	-0.059	0.000																									
ACZ	**0.427*****	**0.146***	-0.035	**0.264*****	-0.064	-0.054	0.000																								
DZ	**0.378*****	**0.236****	0.011	**0.349*****	-0.039	-0.040	-0.041	0.000																							
TL	**0.545*****	0.014	-0.056	**0.126***	-0.017	0.001	-0.010	0.058	0.000																						
LF	**0.441*****	0.124	-0.048	**0.243****	-0.063	-0.050	-0.063	-0.034	-0.025	0.000																					
CX	**0.680*****	-0.050	0.086	0.015	**0.168***	**0.192****	**0.171***	**0.260*****	0.038	**0.146***	0.000																				
BJ	**0.628*****	0.026	0.052	**0.140****	0.087	0.093	0.095	**0.188***	0.003	0.089	0.078	0.000																			
SH	**0.663*****	-0.025	0.060	0.022	**0.146***	**0.166****	**0.152***	**0.238****	0.016	0.130	-0.018	0.048	0.000																		
XCH	**0.372*****	**0.252***	-0.035	**0.431*****	-0.088	-0.088	-0.084	-0.081	0.019	-0.081	**0.297****	0.175	**0.259***	0.000																	
LN	**0.525*****	0.069	-0.029	**0.180*****	0.011	0.026	0.016	0.071	-0.031	0.003	0.093	0.059	0.057	0.032	0.000																
TJ	0.503	-0.948	-0.559	-0.810	-0.393	-0.361	-0.388	-0.220	-0.647	-0.432	-1.000	-0.564	-0.839	-0.367	-0.622	0.000															
ZJ	**0.405*****	**0.787*****	0.556	**0.859*****	**0.453*****	**0.429*****	**0.444*****	**0.345*****	**0.635*****	**0.475*****	**0.808*****	**0.742*****	**0.787*****	**0.428*****	**0.637*****	0.788	0.000														
XC	**0.329*****	**0.632*****	**0.364*****	**0.722*****	**0.258***	**0.242****	**0.252***	**0.155***	**0.450*****	**0.279****	**0.655*****	**0.583*****	**0.632*****	0.179	**0.444*****	0.519	0.014	0.000													
HS	**0.318*****	**0.624*****	**0.358*****	**0.713*****	**0.254****	**0.237****	**0.249****	**0.153****	**0.443*****	**0.274*****	**0.647*****	**0.575*****	**0.624*****	0.168	**0.436*****	0.501	0.020	-0.062	0.000												
JJ	**0.307*****	**0.538*****	**0.268*****	**0.633*****	0.170	0.156	0.165	0.080	**0.350*****	**0.187***	**0.561*****	**0.488*****	**0.538*****	0.078	**0.342*****	0.341	0.073	-0.048	-0.048	0.000											
YY	**0.303*****	**0.490*****	**0.220***	**0.590*****	0.118	0.105	0.113	0.039	**0.296*****	**0.139***	**0.517*****	**0.427*****	**0.491*****	0.031	**0.291****	0.261	0.106	-0.027	-0.024	-0.047	0.000										
XY	**0.288*****	**0.571*****	**0.320****	**0.657*****	**0.220****	**0.203****	**0.213****	**0.125***	**0.397*****	**0.239****	**0.594*****	**0.519*****	**0.571*****	0.128	**0.384*****	0.385	0.019	-0.049	-0.044	-0.039	-0.036	0.000									
NY	**0.354*****	**0.693*****	**0.442*****	**0.775*****	**0.338*****	**0.318*****	**0.333*****	**0.224****	**0.524*****	**0.360*****	**0.716*****	**0.647*****	**0.693*****	**0.273****	**0.519*****	0.623	0.010	-0.032	-0.030	0.000	0.022	-0.023	0.000								
WZ	**0.324*****	**0.598*****	**0.321*****	**0.692*****	**0.212****	**0.189***	**0.208****	0.114	**0.405*****	**0.237*****	**0.625*****	**0.535*****	**0.597*****	0.127	**0.400*****	0.466	0.062	-0.039	-0.042	-0.047	-0.049	-0.047	-0.012	0.000							
SL	**0.456*****	**0.853*****	**0.641*****	**0.918*****	**0.546*****	**0.523*****	**0.536*****	**0.440*****	**0.717*****	**0.565*****	**0.873*****	**0.815*****	**0.853*****	**0.563*****	**0.726*****	0.902	-0.018	0.096	**0.102***	**0.161****	**0.207****	**0.092**	**0.082*****	**0.170*****	0.000						

PY	0.330***	0.322***	0.066	0.434***	-0.004	-0.008	-0.004	-0.047	0.128	0.009	0.348***	0.266**	0.323***	-0.070	0.135	-0.050	0.261***	0.077	0.075	0.015	-0.020	0.055	0.146	0.042	0.361***	0.000					
CD	0.323***	0.588***	0.330***	0.678***	0.219***	0.201**	0.216**	0.135	0.407***	0.247***	0.614***	0.526***	0.589***	0.139	0.400***	0.433	0.044	-0.035	-0.026	-0.021	-0.037	-0.032	0.004	-0.034	0.141*	0.061	0.000				
QJ	0.320***	0.417***	0.142*	0.524***	0.053	0.045	0.049	-0.010	0.216	0.069	0.444***	0.358***	0.417***	-0.028	0.215*	0.134	0.179**	0.013	0.012	-0.031	-0.052	0.002	0.083	-0.013	0.283***	-0.049	0.006	0.000			
XX	0.340***	0.400***	0.133	0.511***	0.038	0.020	0.038	-0.007	0.197	0.061	0.433***	0.317***	0.402***	-0.038	0.203*	0.113	0.232***	0.066	0.063	0.011	-0.032	0.038	0.133	0.007	0.343***	-0.037	0.028	-0.045	0.000		
WN	0.298***	0.528***	0.226*	0.643***	0.123	0.112	0.119	0.037	0.315	0.142	0.557***	0.471***	0.529***	0.029	0.303**	0.269	0.100	-0.055	-0.052	-0.071	-0.070	-0.046	0.005	-0.050	0.211*	-0.023	-0.041	-0.061	-0.018	0.000	
SY	0.271	0.378	-0.095	0.629	-0.200	-0.194	-0.223	-0.259	0.020	-0.198	0.437	0.280	0.362	-0.311	-0.003	-1.000	0.369*	0.014	0.011	-0.110	-0.162	-0.065	0.158	-0.018	0.577*	-0.255	-0.023	-0.228	-0.187	-0.183	0.000

Supplementary Table S3. Analysis of molecular variance (AMOVA) for mtDNA (correspond to HJ, PC and CC, which defined by SAMOVA) and ITS data.

Genes	Source of variation	d.f	Sum of squares	Percentage of variation	Fixation indices
COI+CYTB+ND5	Two level				
	Among populations	30	639.787	29.360	
	Within populations	388	1252.970	70.640	F_{ST} 0.294***
	Three levels				
	Among groups	2	494.310	39.050	F_{CT} 0.390***
	Among populations/within groups	28	145.476	2.650	F_{SC} 0.043**
	Within populations	388	1252.970	58.300	F_{ST} 0.420***
5.8S+ITS2+28S	Two level				
	Among populations	30	36.560	3.030	
	Within populations	375	324.669	96.970	F_{ST} 0.030***

*P<0.05; **P<0.02; ***P<0.001.

Supplementary Table S4. Haplotype frequency by population based on ITS sequences of *Adelphocoris suturalis*.

Haplotype frequency	Haplotype frequency by population																														
	HJ	ZHZ	LX	GY	SH	BB	JYC	ACZ	DZ	TL	LF	CX	BJ	LN	TJ	XCH	ZJ	XC	HS	JJ	YY	XY	NY	WZ	SL	PY	CD	QJ	XX	WN	SY
Hap 1	2																														
Hap 2	3	1		1						1				1		1			2	1		1									
Hap 3	1																														
Hap 4	3												1	1		1	1														
Hap 5	2	1	6	2	2	7	2	4	8	5	6	3	3	1	1	1	5	6	4	5	3	4	3	1	3	8	4	2	6	1	1
Hap 6	2																														
Hap 7	1																														
Hap 8		7	1	3	1	4	9	5	2	3	1	6	3			1	3	3	3	4	5	3	5	4	5	3	2	4	5	3	
Hap 9		1																													
Hap 10		2					2			1	1			4			1					1			2		2			2	
Hap 11		1																													
Hap 12		1																													
Hap 13		1																													
Hap 14			1																												
Hap 15			1		1					1		1						1		1						1	1	1	1		
Hap 16			1																												
Hap 17			2																												
Hap 18			1																					1							
Hap 19			1																								1				
Hap 20			1																												
Hap 21				1																		1								1	
Hap 22				3	1													1			1										
Hap 23				1																											
Hap 24				1																											

Hap 25	1														
Hap 26	1	1													
Hap 27			1												
Hap 28			1												
Hap 29			1												
Hap 30			1												
Hap 31				1											
Hap 32					1										
Hap 33					1										
Hap 34					1										
Hap 35					1										
Hap 36					1										
Hap 37					1										
Hap 38						1			1	1	1				1
Hap 39						1									
Hap 40						1						1	1	1	
Hap 41		1				1	1								
Hap 42						1									
Hap 43							1								
Hap 44							1								
Hap 45							1								
Hap 46								1							
Hap 47								1							
Hap 48								1							
Hap 49								1							
Hap 50								1					1		
Hap 51								1							
Hap 52									2						

Hap 53		1							2
Hap 54			1						
Hap 55			1						
Hap 56			1						
Hap 57			1						
Hap 58			2						
Hap 59	1								
Hap 60	1								
Hap 61	1								
Hap 62	1								
Hap 63	1								
Hap 64	1								
Hap 65	1								
Hap 66					1				
Hap 67				1					
Hap 68						1			
Hap 69						1			
Hap 70						1			
Hap 71						1			
Hap 72						1			
Hap 73							1		
Hap 74							1		
Hap 75							1		
Hap 76							1		
Hap 77								1	
Hap 78								1	
Hap 79								1	
Hap 80								1	

Hap 81	1						
Hap 82		1					
Hap 83		1					
Hap 84		1					
Hap 85		1					
Hap 86			1				
Hap 87			1				
Hap 88			1				
Hap 89				1			
Hap 90				1			
Hap 91				1			
Hap 92				1			
Hap 93				1			
Hap 94					1		
Hap 95					1		
Hap 96					1		
Hap 97					1		
Hap 98					1		
Hap 99					1		
Hap 100						1	
Hap 101						1	
Hap 102						1	
Hap 103						1	
Hap 104						1	
Hap 105						1	
Hap 106						1	
Hap 107							1
Hap 108							1

Hap 109	1						
Hap 110	1						
Hap 111	1						
Hap 112		1					
Hap 113		1					
Hap 114			1				
Hap 115			1				
Hap 116			1				
Hap 117			1				
Hap 118				1			
Hap 119				1			
Hap 120				1			
Hap 121				1			
Hap 122				1			
Hap 123				1			
Hap 124					1		
Hap 125					1		
Hap 126					1		
Hap 127						1	
Hap 128						1	
Hap 129						1	
Hap 130							1

Supplementary Table S5. Genetic diversity statistics for *Adelphocoris suturalis* from each geographic population based on ITS data.

**Population**	**N**	***S***	**Ht**	***Hd***	**π**	**K**
HJ	14	8	7	0.901	0.0016	2.022
ZHZ	15	8	8	0.790	0.0014	1.829
GY	14	11	9	0.923	0.0020	2.539
JYC	14	5	4	0.582	0.0009	1.176
ACZ	15	10	8	0.848	0.0015	1.943
TL	15	12	9	0.876	0.0019	2.514
CX	14	5	6	0.791	0.0009	1.187
BJ	14	10	9	0.923	0.0018	2.297
SH	14	15	13	0.989	0.0020	2.659
XCH	5	4	5	1.000	0.0015	2.000
LN	9	6	6	0.833	0.0021	2.778
NY	14	12	8	0.857	0.0016	2.099
WZ	15	18	11	0.933	0.0026	3.410
SL	15	10	8	0.867	0.0015	1.905
CD	15	10	10	0.923	0.0017	2.229
WN	10	10	7	0.911	0.0021	2.756

LX	15	9	9	0.848	0.0013	1.638
BB	15	7	6	0.742	0.0009	1.219
DZ	15	7	7	0.723	0.0009	1.143
LF	14	12	9	0.835	0.0017	2.176
XY	15	11	10	0.914	0.0015	1.962
ZJ	15	12	9	0.876	0.0016	2.095
XC	15	8	8	0.829	0.0011	1.467
HS	15	8	9	0.905	0.0013	1.657
JJ	15	9	8	0.848	0.0012	1.581
YY	14	9	8	0.857	0.0016	2.044
PY	15	5	6	0.705	0.0009	1.181
QJ	13	10	9	0.910	0.0015	1.923
XX	15	6	6	0.761	0.0009	1.200
SY	2	1	2	1.000	0.0008	1.000
TJ	1	-	1	-	-	-

S, number of segregating sites; Ht, the number of haplotypes; *Hd*, haplotype diversity; π, nucleotide diversity; K, average number of nucleotide difference.

Supplementary Table S6. Migration parameters (M and θ) estimates for 31 populations of *Adelphocoris suturalis* based on mtDNA data.

Population	θ*i*	HJ	ZHZ	LX	GY	BB	JYC	ACZ	DZ	TL	LF	CX	BJ	SH	XCH	LN	TJ	ZJ	XC	HS	JJ	YY	XY	NY	WZ	SL	PY	CD	QJ	XX	WN	SY	
i		→*i*	→*i*	→*i*	→*i*	→*i*	→*i*	→*i*	→*i*	→*i*	→*i*	→*i*	→*i*	→*i*	→*i*	→*i*	→*i*	→*i*	→*i*	→*i*	→*i*	→*i*	→*i*	→*i*	→*i*	→*i*	→*i*	→*i*	→*i*	→*i*	→*i*	→*i*	Total *i*
HJ	0.00227	-	711.3	752.7	560.0	1000.0	992.0	767.3	871.3	1000.0	990.7	1000.0	**490.7**	1000.0	370.7	909.3	882.0	1000.0	870.0	992.7	861.3	990.7	**586.0**	998.0	997.3	834.7	985.3	1000.0	829.3	657.3	986.0	711.3	25597.9
ZHZ	0.00147	790.0	-	1000.0	1000.0	991.3	972.0	1000.0	1000.0	764.0	1000.0	990.7	1000.0	1000.0	656.7	785.3	376.7	990.0	884.0	1000.0	988.0	1000.0	1000.0	914.7	**1000.0**	773.3	860.0	770.0	977.3	800.0	1000.0	683.3	26967.3
LX	0.02940	672.7	564.0	-	1000.0	313.3	1000.0	1000.0	218.0	610.0	997.3	991.3	**428.0**	733.3	999.3	1000.0	93.3	996.7	1000.0	1000.0	1000.0	1000.0	**331.3**	992.0	1000.0	256.7	910.0	998.0	996.7	990.0	204.0	1000.0	23295.9
GY	0.00187	992.7	982.7	378.7	-	**998.7**	1000.0	998.0	1000.0	1000.0	998.0	1000.0	1000.0	1000.0	998.0	1000.0	998.0	882.0	776.0	1000.0	910.7	**565.3**	1000.0	1000.0	992.0	997.3	702.0	975.3	786.7	985.3	1000.0	913.3	27830.7
BB	0.04453	854.0	800.7	993.3	**542.7**	-	853.3	540.0	710.0	888.0	999.3	1000.0	890.0	839.3	544.0	998.0	430.7	958.0	851.3	1000.0	490.0	986.0	555.3	510.0	736.7	609.3	998.7	1000.0	1000.0	**12.0**	1000.0	992.0	23582.6
JYC	0.09873	797.3	1000.0	1000.0	970.7	687.3	-	992.0	1000.0	996.0	898.7	527.3	866.7	680.7	863.3	409.3	995.3	974.7	491.3	852.7	994.7	685.3	986.7	806.0	1000.0	822.0	542.7	990.0	989.3	988.7	688.0	988.7	25485.4
ACZ	0.08300	286.0	580.0	829.3	686.0	1000.0	992.0		990.7	540.0	590.7	**989.3**	538.7	411.3	1000.0	696.0	984.0	**168.7**	738.0	304.0	978.0	770.7	1000.0	996.7	994.0	420.7	**1000.0**	984.7	579.3	667.3	1000.0	863.3	22579.4
DZ	0.07427	995.3	997.3	776.7	731.3	796.0	1000.0	1000.0	-	826.7	327.3	950.0	976.0	644.7	104.0	1000.0	922.0	615.3	905.3	990.7	998.0	**1000.0**	549.3	1000.0	1000.0	999.3	307.3	876.0	**539.3**	841.3	1000.0	185.3	23854.4
TL	0.00140	888.0	1000.0	1000.0	993.3	731.3	994.0	1000.0	1000.0	-	1000.0	980.7	866.7	1000.0	802.7	482.0	1000.0	476.0	997.3	**1000.0**	696.7	1000.0	988.7	1000.0	1000.0	937.3	989.3	981.3	1000.0	990.0	998.7	1000.0	27794.0
LF	0.07613	752.0	740.0	683.3	676.7	1000.0	988.7	1000.0	785.3	880.7	-	1000.0	855.3	534.7	996.7	996.0	846.7	988.7	785.3	1000.0	788.7	998.7	1000.0	982.0	726.0	1000.0	854.7	1000.0	752.7	479.3	1000.0	1000.0	26092.2
CX	0.00147	1000.0	996.7	939.3	748.0	533.3	1000.0	**980.7**	993.3	709.3	504.0	-	1000.0	1000.0	1000.0	830.7	997.3	580.7	999.3	1000.0	999.3	1000.0	998.7	989.3	674.0	1000.0	1000.0	1000.0	987.3	749.3	1000.0	1000.0	27210.5
BJ	0.00140	**1000.0**	1000.0	**1000.0**	1000.0	1000.0	1000.0	1000.0	1000.0	994.0	1000.0	1000.0	-	624.7	1000.0	460.7	1000.0	810.0	992.7	710.0	956.7	1000.0	1000.0	**380.0**	1000.0	837.3	1000.0	988.0	1000.0	1000.0	717.3	1000.0	27471.4
SH	0.00153	998.7	782.0	1000.0	969.3	1000.0	1000.0	997.3	622.7	528.0	1000.0	864.0	1000.0	-	648.7	1000.0	**1000.0**	1000.0	998.7	1000.0	815.3	1000.0	1000.0	1000.0	1000.0	524.7	1000.0	1000.0	995.3	821.3	994.0	784.0	27344.0
XCH	0.09847	700.0	288.7	658.0	840.0	987.3	677.3	1000.0	730.7	727.3	1000.0	998.7	929.3	980.0	-	775.3	1000.0	1000.0	995.3	840.7	362.7	**807.3**	973.3	1000.0	824.7	900.7	**989.3**	998.0	898.0	989.3	360.0	320.0	24551.9
LN	0.00160	1000.0	452.7	1000.0	654.7	1000.0	1000.0	996.0	441.3	458.0	990.7	874.7	988.7	1000.0	1000.0	-	996.0	564.7	1000.0	1000.0	468.7	469.3	989.3	698.0	**1000.0**	1000.0	996.0	916.0	998.0	1000.0	1000.0	1000.0	25952.8
TJ	0.06713	258.7	704.7	324.0	1000.0	702.0	628.7	1000.0	714.0	372.0	1000.0	1000.0	392.0	**218.0**	838.7	847.3	-	862.0	818.0	874.7	466.0	853.3	1000.0	844.0	716.7	903.3	1000.0	991.3	742.0	871.3	1000.0	606.7	22549.4
ZJ	0.05187	1000.0	1000.0	1000.0	766.7	999.3	330.0	**1000.0**	1000.0	980.0	1000.0	552.7	754.0	601.3	983.3	882.7	401.3	-	995.3	670.7	1000.0	983.3	996.7	816.7	874.7	999.3	1000.0	798.0	460.7	824.7	1000.0	996.7	25668.1
XC	0.05667	1000.0	852.0	850.7	829.3	168.0	698.0	991.3	602.0	545.3	995.3	979.3	918.7	794.7	976.7	995.3	1000.0	889.3	-	928.7	980.0	995.3	864.7	998.7	976.0	1000.0	997.3	999.3	494.7	454.7	991.3	994.0	25760.6
HS	0.06747	1000.0	826.7	606.0	914.0	769.3	1000.0	496.0	704.0	**129.3**	881.3	865.3	996.7	664.0	810.7	703.3	1000.0	989.3	999.3	-	996.0	538.0	**953.3**	792.0	992.0	514.0	979.3	336.0	861.3	1000.0	896.7	734.0	23947.8
JJ	0.02193	519.3	588.0	757.3	854.0	362.7	721.3	1000.0	478.0	1000.0	292.7	867.3	478.0	412.7	705.3	489.3	830.0	1000.0	956.0	694.7	-	996.0	999.3	983.3	228.0	866.7	1000.0	886.7	267.3	1000.0	590.0	1000.0	21823.9
YY	0.08387	625.3	374.7	697.3	**992.0**	906.0	973.3	977.3	**233.3**	352.0	656.7	976.7	764.7	1000.0	**92.0**	878.7	1000.0	860.0	1000.0	438.7	1000.0	-	170.0	994.7	826.7	1000.0	653.3	666.7	775.3	834.7	266.0	1000.0	21986.1
XY	0.10000	**998.0**	1000.0	**1000.0**	876.7	250.7	1000.0	1000.0	630.0	608.7	917.3	989.3	870.0	778.7	997.3	992.7	898.7	990.7	994.7	**254.0**	994.0	876.0	-	377.3	627.3	862.0	589.3	736.0	986.0	1000.0	990.0	994.0	25079.4
NY	0.05047	810.0	700.0	552.7	692.7	1000.0	1000.0	978.0	1000.0	986.7	973.3	993.3	**1000.0**	966.7	772.7	991.3	997.3	956.7	997.3	822.7	1000.0	34.7	831.3	-	997.3	393.3	1000.0	984.0	774.0	716.0	1000.0	856.7	25778.7
WZ	0.03607	992.0	**246.7**	996.7	865.3	976.7	996.0	1000.0	678.7	633.3	791.3	634.0	860.7	998.7	1000.0	**233.3**	884.0	998.0	623.3	626.0	1000.0	364.0	1000.0	850.7	-	1000.0	**1000.0**	739.3	424.0	1000.0	**1000.0**	746.7	24159.4

SL	0.07260	998.7	425.3	789.3	773.3	444.7	1000.0	883.3	896.7	702.7	756.0	989.3	996.0	770.0	992.7	729.3	807.3	1000.0	865.3	1000.0	838.7	775.3	454.7	749.3	806.0	-	808.0	989.3	1000.0	1000.0	998.7	990.0	25229.9
PY	0.01553	921.3	538.7	887.3	1000.0	470.0	614.0	**435.3**	530.7	997.3	1000.0	994.0	779.3	590.0	**475.3**	1000.0	979.3	746.7	848.0	1000.0	774.7	984.7	386.0	790.0	**438.0**	799.3	-	885.3	998.0	902.0	986.7	1000.0	23751.9
CD	0.03200	1000.0	807.3	476.0	1000.0	828.0	846.7	985.3	836.7	1000.0	994.7	859.3	866.0	985.3	715.3	1000.0	1000.0	604.0	758.0	582.7	993.3	988.0	992.0	885.3	578.7	522.0	982.0	-	436.0	720.7	1000.0	804.0	25047.3
QJ	0.00440	979.3	990.0	986.0	752.0	656.0	1000.0	887.3	**980.0**	1000.0	982.7	869.3	1000.0	992.0	1000.0	516.0	1000.0	402.7	1000.0	986.7	830.7	1000.0	1000.0	996.7	912.7	978.7	602.0	204.0	-	1000.0	910.7	1000.0	26415.5
XX	0.01640	820.0	873.3	470.7	859.3	**1000.0**	788.7	1000.0	995.3	986.0	931.3	1000.0	982.7	690.0	575.3	872.7	646.7	906.7	1000.0	978.7	810.0	999.3	998.7	986.7	753.3	992.7	1000.0	688.7	988.7	-	1000.0	826.7	26422.2
WN	0.07400	626.7	700.0	624.7	612.0	374.0	994.7	700.0	981.3	994.0	484.7	629.3	657.3	483.3	664.0	824.0	852.0	998.0	1000.0	674.7	980.0	756.0	889.3	518.7	**198.7**	172.0	624.0	728.0	670.7	1000.0	-	999.3	21411.4
SY	0.06947	560.7	978.7	582.7	824.7	822.0	1000.0	1000.0	836.0	1000.0	1000.0	553.3	988.7	701.3	868.7	987.3	998.7	947.3	1000.0	773.3	647.3	994.0	977.3	991.3	496.0	986.7	1000.0	1000.0	556.7	744.0	988.0	-	25804.7

Supplementary Table S7. Number of effective migrants per generation (Nem) for 31 populations of *Adelphocoris suturalis* based on mtDNA data.

Population *i*	θ*i*	HJ →*i*	ZHZ →*i*	LX →*i*	GY →*i*	BB →*i*	JYC →*i*	ACZ →*i*	DZ →*i*	TL →*i*	LF →*i*	CX →*i*	BJ →*i*	SH →*i*	XCH →*i*	LN →*i*	TJ →*i*	ZJ →*i*	XC →*i*	HS →*i*	JJ →*i*	YY →*i*	XY →*i*	NY →*i*	WZ →*i*	SL →*i*	PY →*i*	CD →*i*	QJ →*i*	XX →*i*	WN →*i*	SY →*i*	Total *i*
HJ	0.00227	-	1.6	1.7	1.3	2.3	2.3	1.7	2.0	2.3	2.2	2.3	1.1	2.3	0.8	2.1	2.0	2.3	2.0	2.3	2.0	2.2	1.3	2.3	2.3	1.9	2.2	2.3	1.9	1.5	2.2	1.6	58.1
ZHZ	0.00147	1.2	-	1.5	1.5	1.5	1.4	1.5	1.5	1.1	1.5	1.5	1.5	1.5	1.0	1.2	0.6	1.5	1.3	1.5	1.5	1.5	1.5	1.3	1.5	1.1	1.3	1.1	1.4	1.2	1.5	1.0	39.6
LX	0.02940	19.8	16.6	-	29.4	9.2	29.4	29.4	6.4	17.9	29.3	29.1	12.6	21.6	29.4	29.4	2.7	29.3	29.4	29.4	29.4	29.4	9.7	29.2	29.4	7.5	26.8	29.3	29.3	29.1	6.0	29.4	684.9
GY	0.00187	1.9	1.8	0.7	-	1.9	1.9	1.9	1.9	1.9	1.9	1.9	1.9	1.9	1.9	1.9	1.9	1.6	1.5	1.9	1.7	1.1	1.9	1.9	1.9	1.9	1.3	1.8	1.5	1.8	1.9	1.7	52.0
BB	0.04453	38.0	35.7	44.2	24.2	-	38.0	24.0	31.6	39.5	44.5	44.5	39.6	37.4	24.2	44.4	19.2	42.7	37.9	44.5	21.8	43.9	24.7	22.7	32.8	27.1	44.5	44.5	44.5	0.5	44.5	44.2	1050.1
JYC	0.09873	78.7	98.7	98.7	95.8	67.9	-	97.9	98.7	98.3	88.7	52.1	85.6	67.2	85.2	40.4	98.3	96.2	48.5	84.2	98.2	67.7	97.4	79.6	98.7	81.2	53.6	97.7	97.7	97.6	67.9	97.6	2516.2
ACZ	0.08300	23.7	48.1	68.8	56.9	83.0	82.3	-	82.2	44.8	49.0	82.1	44.7	34.1	83.0	57.8	81.7	14.0	61.3	25.2	81.2	64.0	83.0	82.7	82.5	34.9	83.0	81.7	48.1	55.4	83.0	71.7	1874.1
DZ	0.07427	73.9	74.1	57.7	54.3	59.1	74.3	74.3	-	61.4	24.3	70.6	72.5	47.9	7.7	74.3	68.5	45.7	67.2	73.6	74.1	74.3	40.8	74.3	74.3	74.2	22.8	65.1	40.1	62.5	74.3	13.8	1771.7
TL	0.00140	1.2	1.4	1.4	1.4	1.0	1.4	1.4	1.4	-	1.4	1.4	1.2	1.4	1.1	0.7	1.4	0.7	1.4	1.4	1.0	1.4	1.4	1.4	1.4	1.3	1.4	1.4	1.4	1.4	1.4	1.4	38.9
LF	0.07613	57.2	56.3	52.0	51.5	76.1	75.3	76.1	59.8	67.0	-	76.1	65.1	40.7	75.9	75.8	64.5	75.3	59.8	76.1	60.0	76.0	76.1	74.8	55.3	76.1	65.1	76.1	57.3	36.5	76.1	76.1	1986.4
CX	0.00147	1.5	1.5	1.4	1.1	0.8	1.5	1.4	1.5	1.0	0.7	-	1.5	1.5	1.5	1.2	1.5	0.9	1.5	1.5	1.5	1.5	1.5	1.5	1.0	1.5	1.5	1.5	1.5	1.1	1.5	1.5	40.0
BJ	0.00140	1.4	1.4	1.4	1.4	1.4	1.4	1.4	1.4	1.4	1.4	1.4	-	0.9	1.4	0.6	1.4	1.1	1.4	1.0	1.3	1.4	1.4	0.5	1.4	1.2	1.4	1.4	1.4	1.4	1.0	1.4	38.5
SH	0.00153	1.5	1.2	1.5	1.5	1.5	1.5	1.5	1.0	0.8	1.5	1.3	1.5	-	1.0	1.5	1.5	1.5	1.5	1.5	1.2	1.5	1.5	1.5	1.5	0.8	1.5	1.5	1.5	1.3	1.5	1.2	41.8
XCH	0.09847	68.9	28.4	64.8	82.7	97.2	66.7	98.5	72.0	71.6	98.5	98.3	91.5	96.5	-	76.3	98.5	98.5	98.0	82.8	35.7	79.5	95.8	98.5	81.2	88.7	97.4	98.3	88.4	97.4	35.4	31.5	2417.6
LN	0.00160	1.6	0.7	1.6	1.0	1.6	1.6	1.6	0.7	0.7	1.6	1.4	1.6	1.6	1.6	-	1.6	0.9	1.6	1.6	0.7	0.8	1.6	1.1	1.6	1.6	1.6	1.5	1.6	1.6	1.6	1.6	41.5
TJ	0.06713	17.4	47.3	21.8	67.1	47.1	42.2	67.1	47.9	25.0	67.1	67.1	26.3	14.6	56.3	56.9	-	57.9	54.9	58.7	31.3	57.3	67.1	56.7	48.1	60.6	67.1	66.5	49.8	58.5	67.1	40.7	1513.7
ZJ	0.05187	51.9	51.9	51.9	39.8	51.8	17.1	51.9	51.9	50.8	51.9	28.7	39.1	31.2	51.0	45.8	20.8	-	51.6	34.8	51.9	51.0	51.7	42.4	45.4	51.8	51.9	41.4	23.9	42.8	51.9	51.7	1331.4
XC	0.05667	56.7	48.3	48.2	47.0	9.5	39.6	56.2	34.1	30.9	56.4	55.5	52.1	45.0	55.3	56.4	56.7	50.4	-	52.6	55.5	56.4	49.0	56.6	55.3	56.7	56.5	56.6	28.0	25.8	56.2	56.3	1459.9
HS	0.06747	67.5	55.8	40.9	61.7	51.9	67.5	33.5	47.5	8.7	59.5	58.4	67.2	44.8	54.7	47.5	67.5	66.7	67.4	-	67.2	36.3	64.3	53.4	66.9	34.7	66.1	22.7	58.1	67.5	60.5	49.5	1615.8
JJ	0.02193	11.4	12.9	16.6	18.7	8.0	15.8	21.9	10.5	21.9	6.4	19.0	10.5	9.1	15.5	10.7	18.2	21.9	21.0	15.2	-	21.8	21.9	21.6	5.0	19.0	21.9	19.4	5.9	21.9	12.9	21.9	478.6
YY	0.08387	52.4	31.4	58.5	83.2	76.0	81.6	82.0	19.6	29.5	55.1	81.9	64.1	83.9	7.7	73.7	83.9	72.1	83.9	36.8	83.9	-	14.3	83.4	69.3	83.9	54.8	55.9	65.0	70.0	22.3	83.9	1844.0
XY	0.10000	99.8	100.0	100.0	87.7	25.1	100.0	100.0	63.0	60.9	91.7	98.9	87.0	77.9	99.7	99.3	89.9	99.1	99.5	25.4	99.4	87.6	-	37.7	62.7	86.2	58.9	73.6	98.6	100.0	99.0	99.4	2507.9
NY	0.05047	40.9	35.3	27.9	35.0	50.5	50.5	49.4	50.5	49.8	49.1	50.1	50.5	48.8	39.0	50.0	50.3	48.3	50.3	41.5	50.5	1.8	42.0	-	50.3	19.8	50.5	49.7	39.1	36.1	50.5	43.2	1301.1
WZ	0.03607	35.8	8.9	36.0	31.2	35.2	35.9	36.1	24.5	22.8	28.5	22.9	31.0	36.0	36.1	8.4	31.9	36.0	22.5	22.6	36.1	13.1	36.1	30.7	-	36.1	36.1	26.7	15.3	36.1	36.1	26.9	871.4

SL	0.07260	72.5	30.9	57.3	56.1	32.3	72.6	64.1	65.1	51.0	54.9	71.8	72.3	55.9	72.1	52.9	58.6	72.6	62.8	72.6	60.9	56.3	33.0	54.4	58.5	-	58.7	71.8	72.6	72.6	72.5	71.9	1831.7
PY	0.01553	14.3	8.4	13.8	15.5	7.3	9.5	6.8	8.2	15.5	15.5	15.4	12.1	9.2	7.4	15.5	15.2	11.6	13.2	15.5	12.0	15.3	6.0	12.3	6.8	12.4	-	13.7	15.5	14.0	15.3	15.5	368.9
CD	0.03200	32.0	25.8	15.2	32.0	26.5	27.1	31.5	26.8	32.0	31.8	27.5	27.7	31.5	22.9	32.0	32.0	19.3	24.3	18.6	31.8	31.6	31.7	28.3	18.5	16.7	31.4	-	14.0	23.1	32.0	25.7	801.5
QJ	0.00440	4.3	4.4	4.3	3.3	2.9	4.4	3.9	4.3	4.4	4.3	3.8	4.4	4.4	4.4	2.3	4.4	1.8	4.4	4.3	3.7	4.4	4.4	4.4	4.0	4.3	2.6	0.9	-	4.4	4.0	4.4	116.2
XX	0.01640	13.4	14.3	7.7	14.1	16.4	12.9	16.4	16.3	16.2	15.3	16.4	16.1	11.3	9.4	14.3	10.6	14.9	16.4	16.1	13.3	16.4	16.4	16.2	12.4	16.3	16.4	11.3	16.2	-	16.4	13.6	433.3
WN	0.07400	46.4	51.8	46.2	45.3	27.7	73.6	51.8	72.6	73.6	35.9	46.6	48.6	35.8	49.1	61.0	63.0	73.9	74.0	49.9	72.5	55.9	65.8	38.4	14.7	12.7	46.2	53.9	49.6	74.0	-	73.9	1584.4
SY	0.06947	39.0	68.0	40.5	57.3	57.1	69.5	69.5	58.1	69.5	69.5	38.4	68.7	48.7	60.3	68.6	69.4	65.8	69.5	53.7	45.0	69.1	67.9	68.9	34.5	68.5	69.5	69.5	38.7	51.7	68.6	-	1792.7

Supplementary Table S8. Demographic analysis of *Adelphocoris suturalis* based on mtDNA data.

**Group**	**Fu's Fs**	**Fu's and Li's *D****	**Fu's and Li's *F****
CC	-24.6455***	-5.6047**	-4.3137**
PC	-24.8229***	-5.2670**	-4.0326**
HJ	-0.3440	-0.5307	-0.7963
Whole	-24.0250**	-7.9600**	-5.5380**

CC, PC, HJ groups were defined by SAMOVA (CC, central China group; PC, peripheral China group; HJ, Hokkaido, Japan group).

*P<0.05; **P<0.02; ***P<0.001.

Supplementary Table S9. Sampling information of *Adelphocoris suturalis* from each population.

Population	Detailed locality information	Sample size	Lat. (°N)	Long. (°E)	Ele. (m)	Date
HJ region						
HJ	Hokkaido, Japan	15	43°15'52"	143°33'02"	332	VII. 2013
PC region						
ZHZ	Hangzhou, Zhejiang, China	15	30°18'59"	120°18'36"	13	V. 2013
LX	Lanxi, Zhejiang, China	15	29°11'3"	119°16'22"	67	VIII. 2013
GY	Guiyang, Guizhou, China	15	26°25'4"	106°39'44"	1120	IX. 2013
SH	Suihua, Heilongjiang, China	15	46°10'31"	126°2'5"	160	IX. 2013
BB	Bengbu, Anhui, China	15	33°45'51"	117°26'29"	26	VIII. 2013
JYC	Yancheng, Jiangsu, China	15	33°16'4"	120°27'47"	5	VII. 2013
ACZ	Chizhou, Anhui, China	15	30°19'53"	116°58'9"	18	VIII. 2013
DZ	Dezhou, Shandong, China	15	37°25'4"	116°20'57"	20	VII. 2013
TL	Tieling, Liaoning, China	15	42°37'16"	123°40'50"	82	IX. 2013
LF	Langfang, Hebei, China	15	39°30'11"	116°35'43"	27	VIII. 2012
CX	Cixi, Zhejiang, China	15	30°10'20"	121°13'24"	4	V. 2012
BJ	Haidian, Beijing, China	15	39°54'12"	116°25'17"	181	VII. 2011
LN	Longnan, Gansu, China	10	33°48'10"	106°2'54"	981	IX. 2013

XCH	Xuancheng, Anhui, China	6	30°45'51"	118°52'43"	30	VIII. 2013
TJ	Baodier, Tianjin, China	1	39°23'59"	117°20'44"	4	VII. 2011
CC region						
ZJ	Zhijiang, Hubei, China	15	30°25'40"	111°52'44"	42	V. 2013
XC	Xuchang, Henan, China	15	33°57'44"	113°37'52"	79	VIII. 2013
HS	Hengshui, Hebei, China	15	37°42'15"	115°47'42"	8	IX. 2013
JJ	Jiujiang, Jiangxi, China	15	29°49'36"	116°24'45"	69	V. 2013
YY	Yueyang, Hunan, China	15	29°29'30"	112°34'22"	28	V. 2013
XY	Xiangyang, Hubei, China	15	32°10'34"	112°37'2"	123	VIII. 2013
NY	Nanyang, Henan, China	15	32°56'29"	112°33'50"	121	VIII. 2013
WZ	Wuzhong, Ningxia, China	15	38°1'7"	106°6'29"	1120	IX. 2013
SL	Shangluo, Shanxi, China	15	34°4'12"	110°3'37"	1020	VIII. 2013
PY	Puyang, Henan, China	15	35°35'51"	115°6'42"	48	VII. 2013
CD	Changde, Hunan, China	15	29°38'43"	111°49'13"	44	V. 2013
QJ	Qianjiang, Hubei, China	15	30°29'1"	112°46'30"	40	V. 2012
XX	Xinxiang, Henan, China	15	35°17'13"	113°56'50"	72	IX. 2011
WN	Weinan, Shanxi, China	10	34°47'20"	110°1'4"	344	VIII. 2013
SY	Songyuan, Jilin, China	2	44°16'3"	123°55'54"	157	IX. 2013

Supplementary Table S10. Primers sequences of amplification used in this study.

Gene	Name	Sequence (5'–3')
COI	COI-F	CGAATTAGGAATACCAGGATCATTC
	COI-R	CAAATCCTGGTAGGATTAGAATATA
CYTB	CYTB-F	GACCAAGAAGGATTTCATTATGATG
	CYTB-R	CGTGCTCCAATTCAAGTTAATAAGA
ND5	ND5-F	TAGTCATTCTTATATACAGGATGAT
	ND5-R	GGTAAACCAGATAAAGAAAAAGACG
5.8S+ITS2+28S	28Z	AGACTCCTTGGTCCGTGTTTC
	P1	ATCACTCGGCTCGTGGATCG

Figure S1. Scatter plot showing the correlation of genetic distances (F_{ST}) and geographical distances (km) based on mtDNA data.

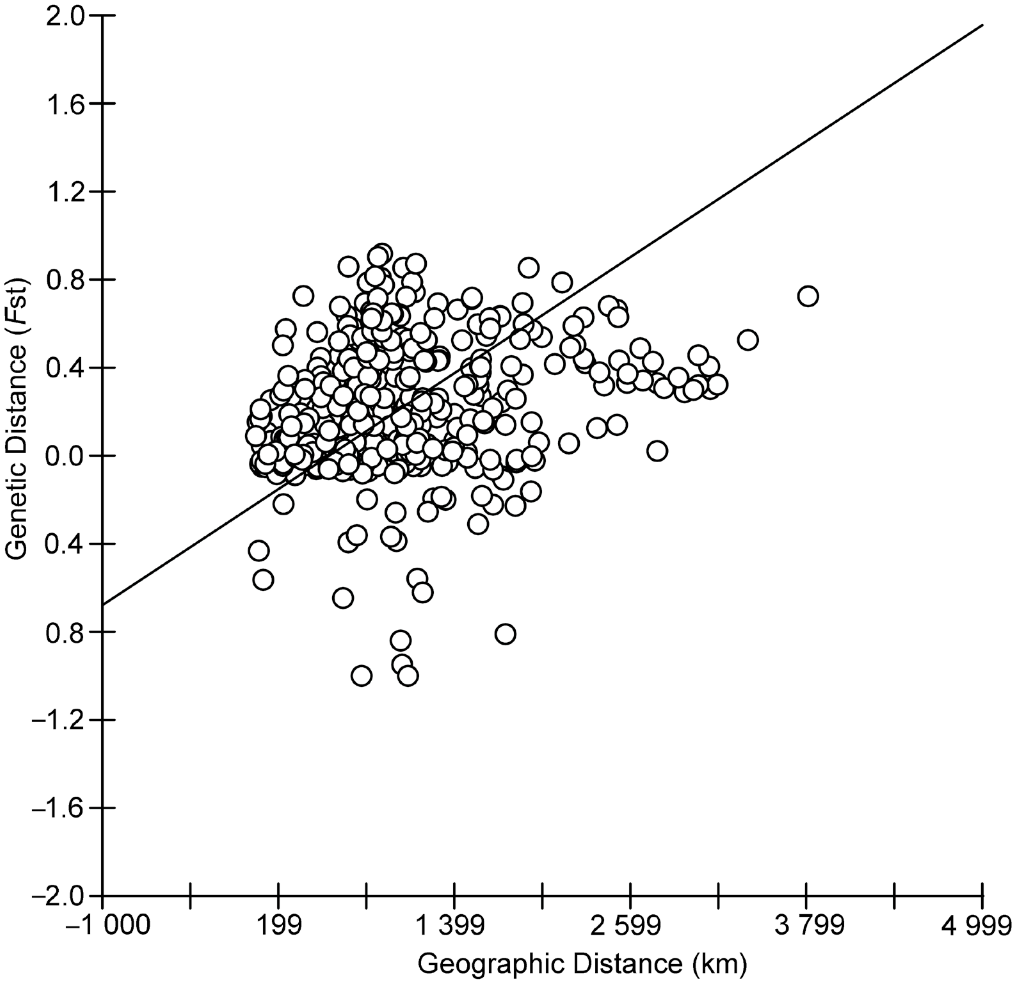

Figure S2. Genetic barriers predicted by BARRER based on ITS data. The genetic barriers are shown in red lines with arrows labeled from 'a' to 'I'. Map was generated from http://ngcc.sbsm.gov.cn/ (Data of access: 18/03/2015).

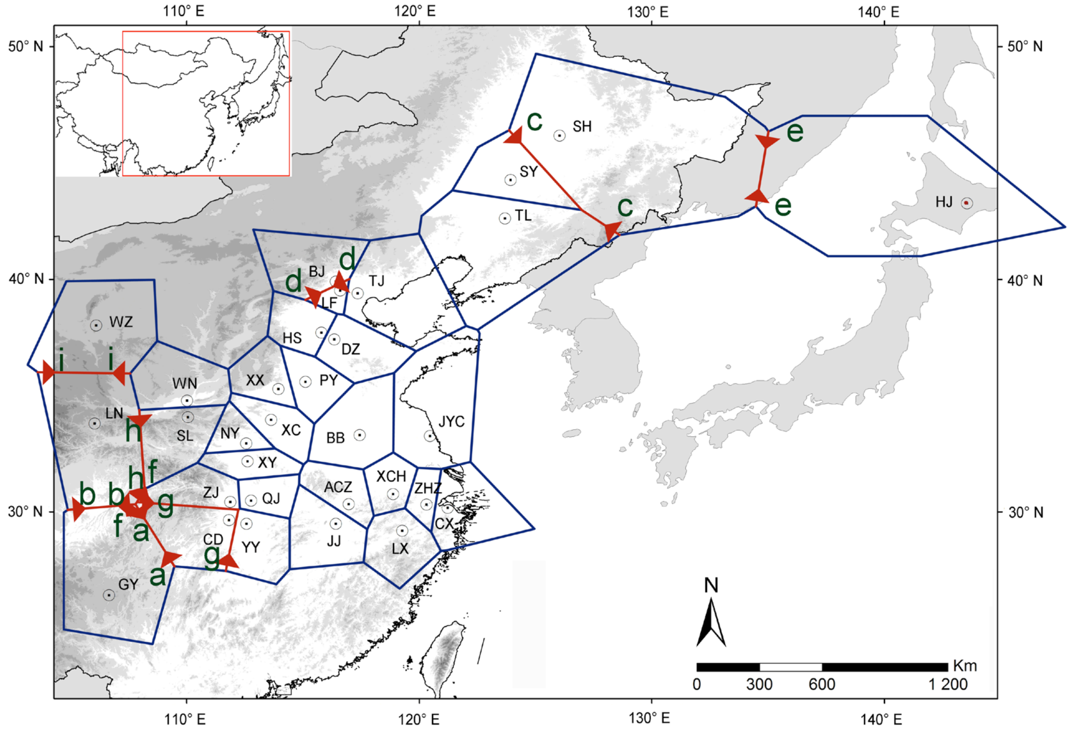

Figure S3. Phylogenetic tree of the haplotypes for *Adelphocoris suturalis* based on mtDNA data. Bayesian posterior probabilities, maximum likelihood bootstrap percentages are shown as branch support (support is only shown if > 0.5/50 for at least one method). When the haplotype was unique to one individual, the population name with a number denoted the haplotype name. When the haplotype was unique to same population, the population name with a letter denoted the haplotype name. When the haplotype was belonged to different populations, the "Hap" with a number denoted the haplotype name. When the haplotype was observed in other two countries, the country name was added in the haplotype name. The illustration of *A. suturalis* was drawn by L.Z.

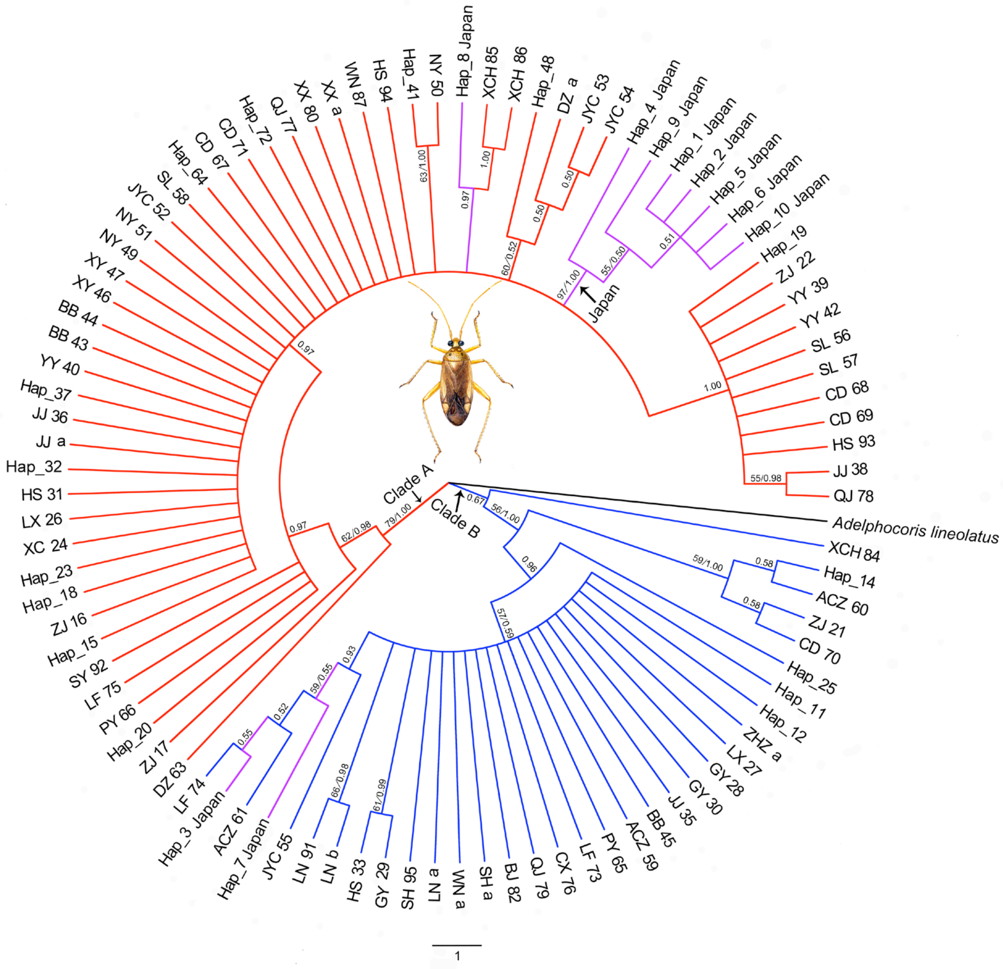

Figure S4. Phylogenetic tree of the haplotypes for *Adelphocoris suturalis* based on ITS data. Bayesian posterior probabilities, maximum likelihood bootstrap percentages are shown as branch support (support is only shown if > 0.5/50 for at least one method). (The annotation is same as Fig. S3).

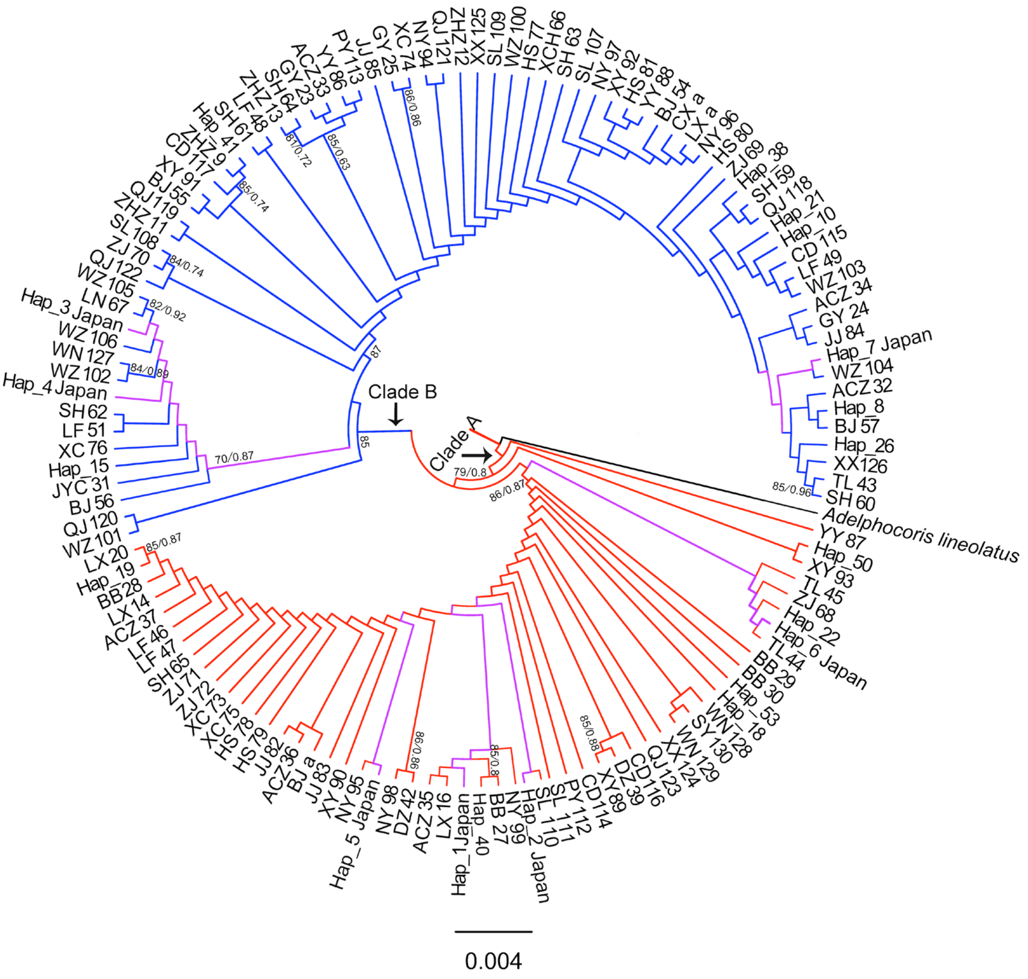

Supplement: Supplementary Information [file srep14009-s1.pdf]
